# Xenografted human microglia display diverse transcriptomic states in response to Alzheimer’s disease-related amyloid-β pathology

**DOI:** 10.1038/s41593-024-01600-y

**Published:** 2024-03-27

**Authors:** Renzo Mancuso, Nicola Fattorelli, Anna Martinez-Muriana, Emma Davis, Leen Wolfs, Johanna Van Den Daele, Ivana Geric, Jessie Premereur, Paula Polanco, Baukje Bijnens, Pranav Preman, Lutgarde Serneels, Suresh Poovathingal, Sriram Balusu, Catherine Verfaillie, Mark Fiers, Bart De Strooper

**Affiliations:** 1 Microglia and Inflammation in Neurological Disorders (MIND) Lab, VIB Center for Molecular Neurology, VIB, Antwerp, Belgium; 2 Department of Biomedical Sciences, University of Antwerp, Antwerp, Belgium; 3 Centre for Brain and Disease Research, Flanders Institute for Biotechnology (VIB), Leuven, Belgium; 4 Department of Neurosciences and Leuven Brain Institute, KU Leuven, Leuven, Belgium; 5 UK Dementia Research Institute at UCL, University College London, London, UK; 6 Department of Development and Regeneration, Stem Cell Biology and Embryology, KU Leuven Stem Cell Institute, Leuven, Belgium

**Keywords:** Cellular neuroscience, Microglia, Alzheimer's disease

## Abstract

Microglia are central players in Alzheimer’s disease pathology but analyzing microglial states in human brain samples is challenging due to genetic diversity, postmortem delay and admixture of pathologies. To circumvent these issues, here we generated 138,577 single-cell expression profiles of human stem cell-derived microglia xenotransplanted in the brain of the *App*^*NL-G-F*^ model of amyloid pathology and wild-type controls. Xenografted human microglia adopt a disease-associated profile similar to that seen in mouse microglia, but display a more pronounced human leukocyte antigen or HLA state, likely related to antigen presentation in response to amyloid plaques. The human microglial response also involves a pro-inflammatory cytokine/chemokine cytokine response microglia or CRM response to oligomeric Aβ oligomers. Genetic deletion of *TREM2* or *APOE* as well as *APOE* polymorphisms and *TREM2*^*R47H*^ expression in the transplanted microglia modulate these responses differentially. The expression of other Alzheimer’s disease risk genes is differentially regulated across the distinct cell states elicited in response to amyloid pathology. Thus, we have identified multiple transcriptomic cell states adopted by human microglia in a multipronged response to Alzheimer’s disease-related pathology, which should be taken into account in translational studies.

## Abstract (web summary)

Human microglia transplanted in the mouse brain mount a multipronged response to amyloid-β pathology, displaying unique transcriptional states. Alzheimer’s disease risk genes are differentially regulated across cell states and profoundly alter microglial function.

## Main

Microglia are a central part of the cellular response in Alzheimer’s disease (AD) pathogenesis^1,2^, in particular in the early response to amyloid-β (Aβ) pathology^3–6^. Murine microglia transit from a homeostatic state toward ‘reactive’ disease-associated microglia (DAM), also called activated response microglia (ARM) or neurodegenerative microglia (MGnD)^4,5^. This response is partially Triggering receptor expressed in myeloid cells 2 (TREM2) and Apolipoprotein E (APOE)-dependent and elicited by Aβ plaques in mice^3,4,7^. Ageing, tauopathy, amyotrophic lateral sclerosis^4,5,8,9^, apoptotic neurons^5^, Danish amyloid^10^ and even peripheral lipid dyshomeostasis^11^ can elicit a similar response, suggesting that the DAM state is a generic response of mouse microglia to damage. However, it is somewhat controversial as to what extent the DAM response is conserved in the human brain^12,13^. Mouse and human microglia are evolutionarily divergent, for instance, regarding the expression of relevant AD genetic risk^7,14,15^.

In addition, the microglial cell states reported by postmortem samples are inconsistent. Mathys et al.^16^ found 22 genes upregulated in microglia from patients with AD (only five overlapping with the DAM signature). Grubman et al.^17^ reported eight genes, whereas Zhou et al.^18^ identified 11 DAM genes enriched in patients with AD compared with controls. Del-Aguila et al.^19^ analyzed single-nucleus transcriptomes from three patients with AD and were unable to recapitulate an activation signature. A large study by Gerrits et al.^20^ analyzed 482,472 single nuclei from nondemented control and AD brains and indicated several distinct transcriptomic states of microglia encompassing DAM-like (*ITGAX*, *SPP1*) and pro-inflammatory (*IL1B*, *NFKB1*) states in AD. A more recent study by Sun et al.^21^ analyzed 194,000 single nuclei from 443 AD and healthy subjects, reporting 12 distinct microglial transcriptional states with clear pro-inflammatory profiles (*IL1B*, *CCL3*), but no clear enrichment for DAM signatures. The lack of congruency in these human postmortem brain studies can be partially explained by technical issues, such as postmortem time^12^, or intrinsic lesser resolution of single-nuclei sequencing approaches^13^, heterogeneity in pathological samples or technical shortcomings including sample size. Additionally, there are inherent limitations to postmortem studies as they reflect only terminal stages of a complex, decade-long disease process, which often encompasses additional concomitant neuropathologies (Tau, TDP-43, vascular dementia or alpha-synuclein).

Here, we circumvent these multiple drawbacks by using a xenotransplantation model where we engrafted stem cell-derived microglia in the brain of the *App*^*NL-G-F*^ model of amyloid pathology and of wild-type (WT) controls^14^. We provide a full transcriptomic characterization of the different cell states adopted by human microglia in response to Aβ pathology, including the elusive component of soluble Aβ oligomers (Aβo) that have been linked to neuroinflammation, neurodegeneration and cognitive impairment^22–25^. We show that human microglia react very differently from mouse microglia to Aβ pathology, with several branches all differentially enriched in AD risk genes, and which can be selectively disturbed by introducing the *TREM2* risk variant or *APOE* polymorphisms.

## Results

### Response of human microglia to Aβ pathology

**Fig. 1:**
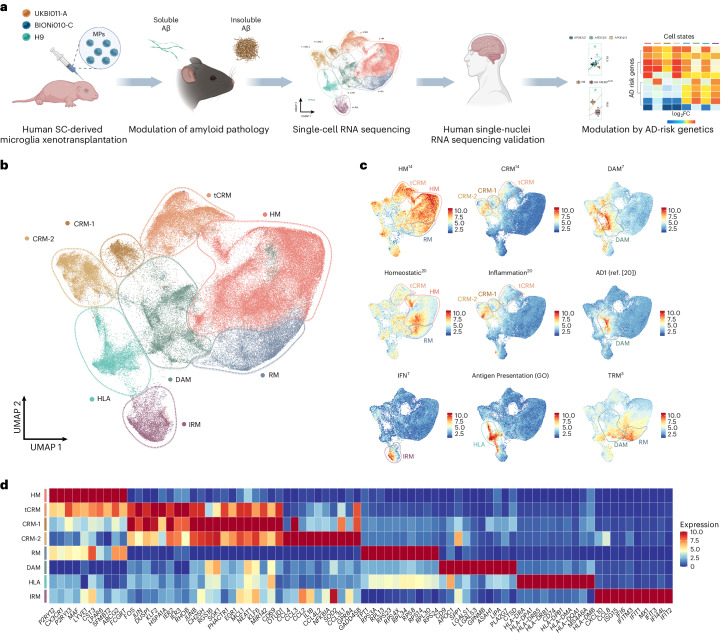
Human microglia display a complex, heterogeneous response to Aβ pathology. **a**, Experimental design used in this study. **b**, UMAP plot visualizing 138,577 single xenografted human microglial cells sorted from mouse brain (CD11b^+^hCD45^+^) ~6 months after transplantation. Cells are colored according to clusters identified: HM, RM, DAM, IRM, HLA, CRM-1 and CRM-2, and tCRM. The assignment of different clusters to distinct cell types or states is based on previous experimental data from our and other laboratories^3,4,7,14^ (see **c**,**d** and Extended Data Figs. 3 and 4). **c**, UMAP plots as in **b**, colored by the combined level of expression of groups of genes that characterize distinct microglial transcriptional states. **d**, Top ten most differentially expressed genes in each cluster (normalized expression scaled by gene is shown). FC, fold change; GO, gene ontology; MP, microglial precursor; SC, stem cell.

We transplanted microglial precursors in the mouse brain according to the MIGRATE (microglia in vitro generation refined for advanced transplantation experiments) protocol^26^. We used three different host genetic backgrounds, that is *Rag2*^−/−^ *Il2rγ*^−/−^ *hCSF1*^*KI*^ *App*^*NL-G-F*^ (with progressive Aβ plaque accumulation from 3 months after birth, thereafter called *App*^*NL-G-F*^), *Rag2*^−/−^ *Il2rγ*^−/−^ *hCSF1*^*KI*^ *App*^*hu/hu*^ mice (humanized *App* controls^27^ for the *App*^*NL-G-F*^ mice that we called *App*^*WT*^ in the current manuscript) and *Rag2*^−/−^ *Il2rγ*^−/−^ *hCSF1*^*KI*^ *App*^*NL-G-F*^ *ApoE*^−/−^ (*App*^*NL-G-F*^ *ApoE*^−/−^) mice (Fig. 1a). The *App*^*WT*^ mice are a better control than the initially proposed *App*^*NL*^ mouse, as discussed by Serneels et al.^27^. We also studied stem cell-derived microglia from three different human genetic backgrounds, that is, UKBIO11-A, BIONi010-C and H9 (Methods and Table 1). Microglia were transplanted in 4-day-old (P4) neonates and isolated at 6–7 months after transplantation by fluorescence-activated cell sorting (FACS) (CD11b^+^hCD45^+^; Fig. 1a and Supplementary Fig. 1b). We obtained high-quality single-cell transcriptomes from 138,577 microglia across 106 mice, excluding CNS-associated macrophages (CAMs), proliferative cells, other myeloid cells, and low-quality cells and doublets (Supplementary Figs. 2 and 3).

**Table 1:** Summary of cell lines used in this study

Name of line	Genotype	Source	Citation
UKBi011-A	APOE e4/e4	Bioneer, EBiSC	RRID:CVCL_LE34
UKBi011-A-1	APOE KO/KO	Bioneer, EBiSC	RRID:CVCL_RM82
UKBi011-A-2	APOE e2/e2	Bioneer, EBiSC	RRID:CVCL_VN45
UKBi011-A-3	APOE e3/e3	Bioneer, EBiSC	RRID:CVCL_RX83
BIONi010-C-2	APOE e3/KO	Bioneer, EBiSC	RRID:CVCL_II81
BIONi010-C-3	APOE KO/KO	Bioneer, EBiSC	RRID:CVCL_II82
BIONi010-C-4	APOE e4/KO	Bioneer, EBiSC	RRID:CVCL_II83
BIONi010-C-6	APOE e2/KO	Bioneer, EBiSC	RRID:CVCL_II85
H9 (WA09)	APOE e3/e4	WiCell Research Institute	RRID:CVCL_9773
H9-iCas9	APOE e3/e4	VIB (Vlaams Instituut voor Biotechnologie) Center for Brain and Disease Research	
H9-TREM2^−/−^	TREM2^−/−^	Katholieke Universiteit Leuven (KUL) Stem cell Institute	Claes et al.^46^
H9-TREM2^R47H^	TREM2^R47H^	KUL Stem Cell Institute	Claes et al.^46^

We identified five distinct microglial clusters, which we annotated as Homeostatic (HM), Cytokine response-1 and -2 (CRM-1 and -2), Interferon response (IRM), Disease-associated response (DAM) and Antigen-presenting response (HLA), as well as two intermediate clusters which we named Ribosomal microglia (RM), enriched in ribosomal genes, and Transitioning CRM (tCRM) microglia, which show high levels of homeostatic genes but also express CRM markers (Fig. 1b–d, Supplementary Fig. 4 and Table 1). The DAM and IRM clusters have similar counterparts in mice^3,4^ (Supplementary Fig. 4b). Using existing datasets^3,4^, we observed that only 20–25% of the previously described mouse DAM signature overlapped with the transcriptome of human xenografted microglia (Extended Data Fig. 3d and Supplementary Table 2). In addition, and contrary to mouse cells, human microglia do not seem to display the DAM1/DAM2 duality^4^. Although CRM and HLA cell states have been described before in mouse models of trauma and as a response to neuronal death^28^, they were not identified before in mouse microglia derived from mouse models of Aβ pathology, including *App*^*NL-G-F*^ (ref. ^3^). CRM is defined by the upregulation of genes encoding cytokines and chemokines (*CCL4*, *CCL3*, *CCL42L*, *CCL3L1*, *GADD45B*, *CCL2*, *CH25H*, *IL1B*, *ZFP36*, *SGK1*), while HLA encompasses *HLA-DRA*, *HLA-DPA1*, *HLA-DRB5*, *CD74*, *HLA-DPB1*, *HLA-DMA*, *MS4A6A* and *HLA-DMB* expression (Fig. 1d and Supplementary Fig. 4). Interestingly, whereas the DAM profile shows a clear downregulation of homeostatic microglial genes, these are much less downregulated in CRM, HLA and IRM clusters (Fig. 1d).

**Extended Data Fig. 1:**
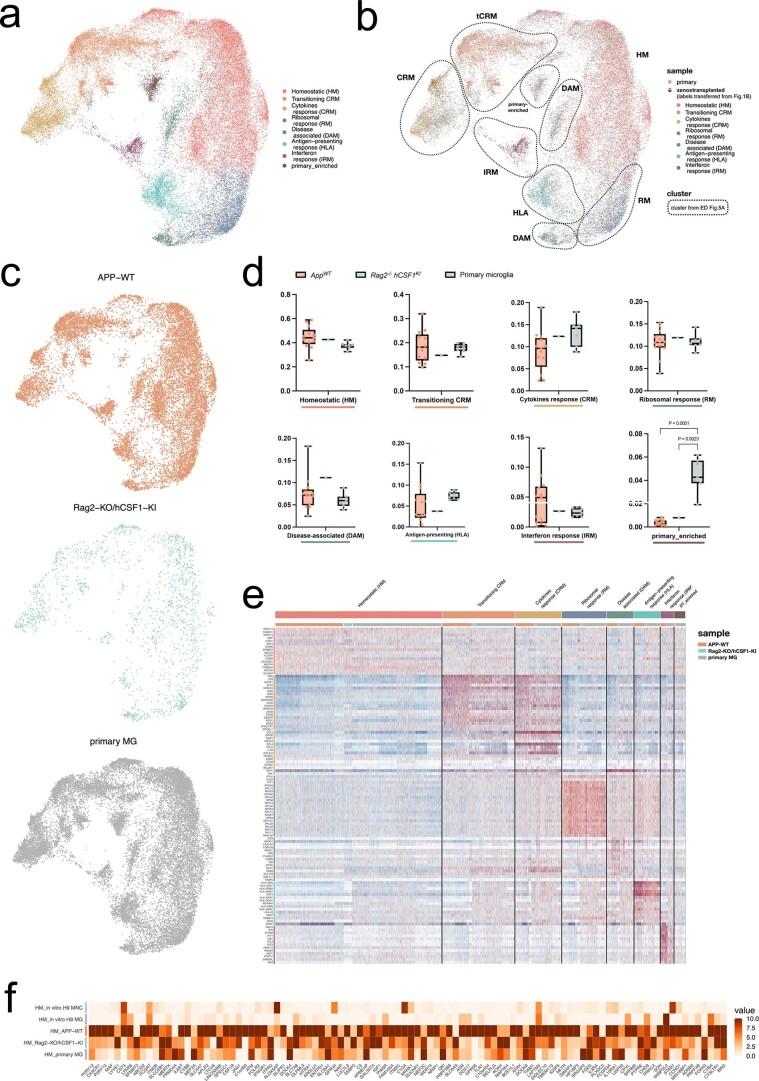
Comparison of human microglia transplanted in *App*^*WT*^ and *Rag2*^−/−^*hCSF*^*KI*^ mice, and primary microglia isolated from human surgical resections published before^14^. **a**, UMAP plot of the 39,538 cells passing quality control, coloured by annotated cell states after removal of CAMs, other myeloid, low quality and proliferating clusters. **b**, UMAP plots as in **a**, coloured by sample. c, UMAP plots as in a, split between the three experimental groups. **d**, Box plot showing proportion of cells across all identified clusters (box plots are limited by lower and upper quartiles and midline indicates median; whiskers show minimum to maximum value. Each dot represents a single replicate, n = 13 for *App*^*WT*^, n = 1 for *Rag2*^−/−^*/hCSF1*^*KI*^, n = 7 for primary MG. One-way ANOVA with Tukey’s multiple comparisons as post-hoc test, alpha = 0.05, significance was set as P-adjusted value < 0.05). **e**, Heatmap displaying the top20 most upregulated genes in each cluster as in **a. f**, Heatmap depicting the expression levels of homeostatic microglial markers across all experimental groups (normalized expression scaled by gene is shown).

As a quality control, we compared the transcriptomic profiles of cells transplanted into *App*^*WT*^ mice (*n* = 10, 3 independent cell lines) with those previously obtained from microglia transplanted into the brain of *Rag2*^−/−^ *Il2rγ*^−/−^ *hCSF1*^*KI*^, and from primary human microglia freshly isolated from surgical resections^14^. As expected, we found a vast overlap between transplanted and primary cells, with all clusters represented to similar extents in the different experimental groups, confirming that the engrafted cells acquire a mature transcriptomic profile resembling that of human adults. Of note, a small cluster that represented 4% of the dataset was enriched only in the primary human cells (Extended Data Fig. 1 and Supplementary Table 3).

**Fig. 2:**
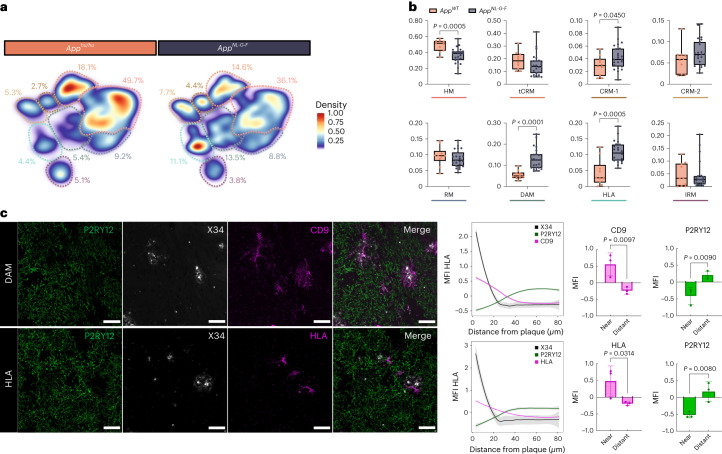
Multipronged response of human microglia upon exposure to Aβ. **a**, Density plots displaying the average distribution of human microglia transplanted in *App*^*NL-G-F*^ (*n* = 23) and *App*^*WT*^ (*n* = 10) mice (density is normalized to sample size). **b**, Distribution and proportion of cells across all identified clusters (box plots are limited by lower and upper quartiles and midline indicates median; whiskers show minimum to maximum values. Each dot represents a single mouse. Unpaired *t*-test with Welch’s correction, two-tailed, alpha = 0.05, significance was set as *P* < 0.05). **c**, Representative images and quantification of human microglia engrafted in the brain of *App*^*NL-G-F*^ mice at 6 months of age, labeled with human-specific antibodies for P2RY12 (HM microglia), CD9 (DAM), HLA-DR/DQ (HLA microglia), as well as X-34 for Aβ plaques. The shift in immunofluorescent signal in the proximity of Aβ plaques was performed using a modified Sholl analysis where the fluorescent intensity of microglial markers P2RY12, CD9 and HLA was measured through concentric rings (annuli) of increasing diameter surrounding the X-34 plaque center. Intensities of each channel were scaled for comparison using *z*-score normalization. Intensity over distance (µm) was plotted using Loess nonparametric regression in R with estimated standard error for each predicted value. For comparison of intensities near and distant from the plaque center, the means of the inner and outer three annuli were independently calculated (near, 0–10 mm, distant, 70–80 mm from the plaque; *n* = 3 per group; bar plots represent mean ± s.e.m.; unpaired *t*-test, one-tailed, alpha = 0.05, significance was set as *P* < 0.05). Scale bars, 50 μm. MFI, median fluorescent intensity.

**Extended Data Fig. 2:**
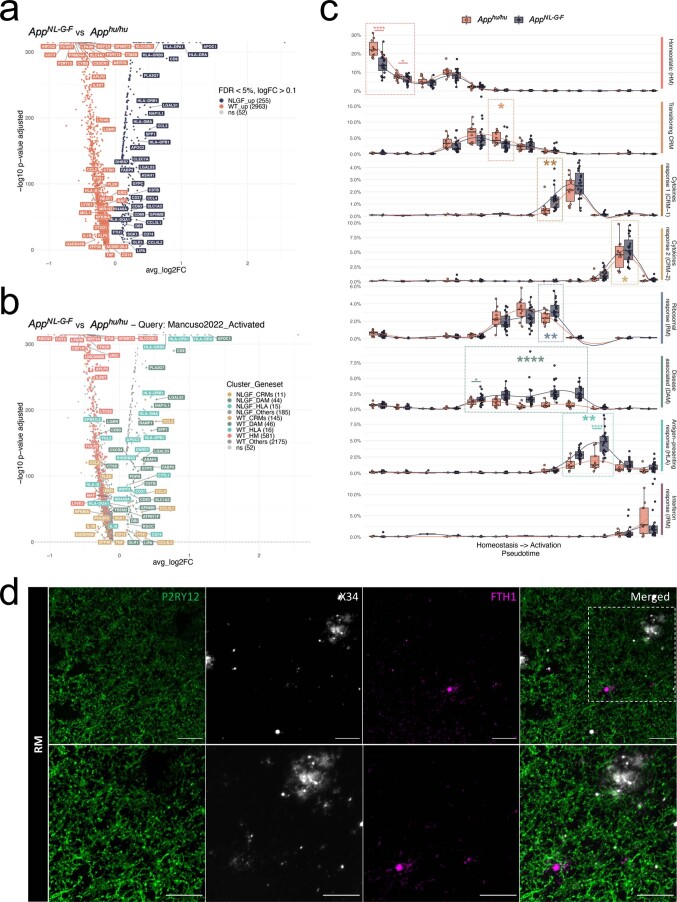
Extended analysis of the cell state transition in *App*^*NL-G-F*^ and *App*^*WT*^ mice. **a**, Volcano plot showing a paired comparison between *App*^*NL-G-F*^ and *App*^*WT*^ mice (Wilcoxon rank-sum test, P-values adjusted with Bonferroni correction based on the total number of genes in the dataset). The number of significant genes per condition is reported in brackets in the legend (Log2 Fold-Change threshold > 0.1). Adjusted p-value threshold < 0.05 (ns = not significant). **b**, Volcano plot showing a paired comparison between *App*^*NL-G-F*^ and *App*^*WT*^ mice (Wilcoxon rank-sum test, P-values adjusted with Bonferroni correction based on the total number of genes in the dataset), coloured by clusters as in Fig. 1 (HM = red squares, CRMs (CRM-1 & CRM-2) = ochre circles, DAM = olive triangles, HLA = cyan diamonds, other markers = grey circles). The number of significant genes per condition is reported in brackets in the legend (Log2 Fold-Change threshold > 0.1). Adjusted p-value threshold < 0.05 (ns = not significant). **c**, Phenotypic trajectory followed by the human microglia obtained by an unbiased pseudotime ordering with *Monocle 3*. Proportion of cells (y-axis) present in each cell state as in Fig. 1a (rows) at different stages of the binned pseudotime trajectory (x-axis) in *App*^*NL-G-F*^ and *App*^*WT*^ mice (box plots are limited by lower and upper quartiles and midline indicates median; whiskers extend from the box to the smallest or largest value no further than 1.5*inter-quartile range. Each dot represents a single mouse, n = 10 for *App*^*WT*^, n = 23 *App*^*NL-G-F*^. Unpaired *t*-test with Welch’s correction, two-tailed, alpha = 0.05, significance was set as P-value < 0.05; *p < 0.05, **p < 0.01, ****p < 0.0001). **d**, Representative images of human microglia engrafted in the brain of *App*^*NL-G-F*^ mice at 6 months of age and labelled with human specific antibodies FTH1 (RM). Human P2RY12 was stained in green, and amyloid-β plaques were stained with X34 in white. Scale bars are 25 μm (top row) and 50 μm (bottom row).

**Extended Data Fig. 3:**
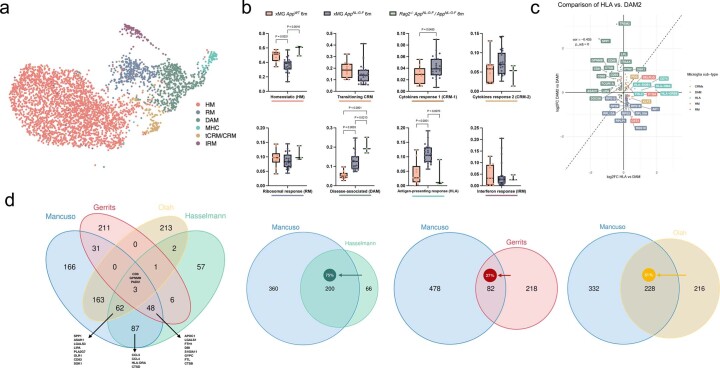
Extended comparison of the response of human and mouse microglia to amyloid pathology. **a**, UMAP plot of the 3,803 mouse microglia isolated from *App*^*NL-G-F*^ and *Rag2*^−/−^ *Il2rγ*^−/−^ *hCSF1*^*KI*^ *App*^*NL-G-F*^ passing quality control, coloured by annotated cell states and after removal of CAMs, other myeloid, low quality and proliferating clusters. **b**, Distribution and proportion of cells across all identified clusters (box plots are limited by lower and upper quartiles and mid line indicates median; whiskers show minimum to maximum value. Each dot represents a single mouse, n = 10 for xMG *App*^*WT*^ 6 m, n = 23 for xMG *App*^*NL-G-F*^ 6 m, n = 2 for *App*^*NL-G-F*^). One-way ANOVA with Tukey’s multiple comparisons as post-hoc test, alpha = 0.05, significance was set as P-adjusted value < 0.05). For cell states that only had 2 groups (tCRM and CRM-1), we applied an unpaired *t*-test with Welch’s correction, alpha = 0.05, two-tailed, significance was set as P-value < 0.05. Note that whereas mouse cells polarize to DAM, HLA and CRM, proportions do not increase. **c**, Correlation analysis of the logFC in DAM2 vs. DAM1 clusters defined by Keren-Shaul et al.^4^ (y-axis) and HLA vs. DAM clusters defined in this study (x-axis). Pearson’s correlation, R = −0.44, differentially expressed genes were adjusted using Bonferroni correction and colored according to clusters in Fig. 1a. **d**, Overlap in the number of genes between our DAM signature and that from mouse systems (KarenShaul DAM and SalaFrigerio ARM), other human microglia xenotransplantation studies (Hasselman DAM), and postmortem single nuclei samples (Gerrits AD1, and Olah DAM). The arrow depicts the direction used to calculate the percentage overlap.

Next, we compared the transcriptomic profiles of human microglia transplanted in *App*^*NL-G-F*^ (*n* = 23) and *App*^*WT*^ (*n* = 10) (three independent cell lines per group) mice to evaluate to what extent different microglial phenotypes were induced by Aβ pathology. DAM, HLA and CRM-1 clusters were significantly enriched in *App*^*NL-G-F*^ compared with *App*^*WT*^ mice (Fig. 2a,b and Extended Data Fig. 2a,b). This is only partially consistent with a previous study by Hasselman et al.^7,29^ (Extended Data Fig. 3d), where DAM but not antigen-presenting (HLA/MHC-II) or chemokine transcriptional profiles were correlated to Aβ pathology in a human chimera model. Nevertheless, the authors used only *n* = 1 mice per group and a different amyloid mouse model. We performed immunostaining using human-specific markers to confirm that both human DAM (CD9) and HLA (HLA-DQ/DR) microglia clustered around Aβ plaques (Fig. 2c), as well as the intermediate RM state (FTH1) (Extended Data Fig. 2d). Expression of various genes of the HLA cluster has been associated with dense cored Aβ plaques in human AD brain, and was detected also in demyelinating regions in multiple sclerosis cases^30^.

**Fig. 3:**
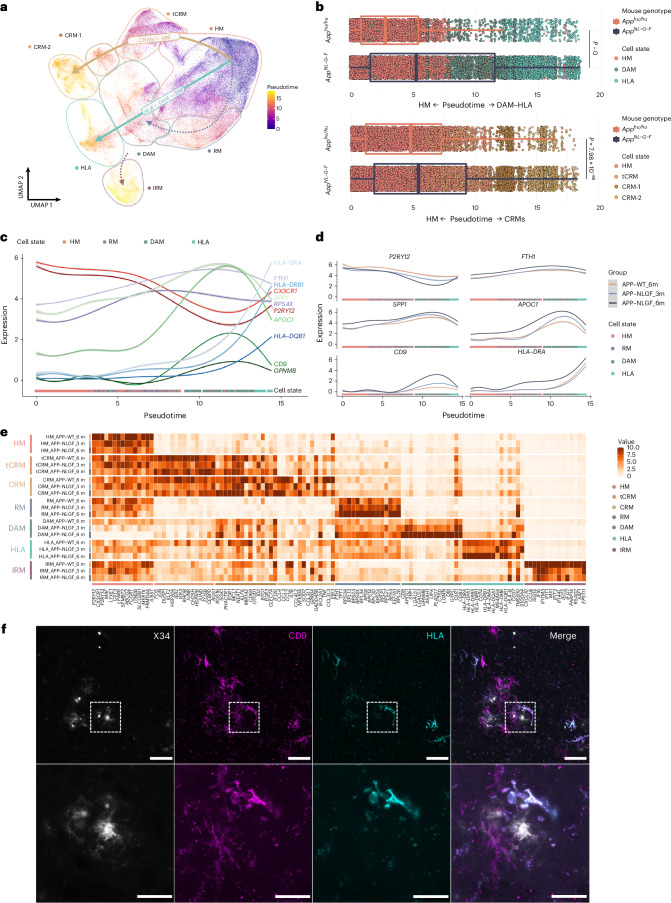
Human microglial transcriptional trajectory in response to Aβ pathology. **a**, Phenotypic trajectory followed by the human microglia after exposure to Aβ in vivo, obtained by an unbiased pseudotime ordering with Monocle 3. **b**, Distribution of cells from different host mouse genetic backgrounds (*y* axis) across the two main transcriptional trajectories, DAM and HLA (top panel) and CRM (bottom panel), colored by clusters shown in **a**. Note the shift in transcriptional states in *App*^*NL-G-F*^ versus *App*^*WT*^ mice (box plots are limited by lower and upper quartiles and midline indicates median; whiskers extend from the box to the smallest or largest value no further than 1.5 × interquartile range. Each dot represents a single cell, *n* = 10 mice (10,663 cells) for *App*^*WT*^, *n* = 23 mice (32,436 cells) for *App*^*NL-G-F*^. Unpaired *t*-test with Welch’s correction, two-tailed, alpha = 0.05, significance was set as *P* < 0.05). **c**,**d**, Expression profile of selected genes across the HM–DAM–HLA pseudotime axis from the entire dataset (**c**), and divided into the different experimental groups: *App*^*WT*^, *App*^*NL-G-F*^ at 3 months and *App*^*NL-G-F*^ at 6 months (**d**). **e**, Top ten most differentially expressed genes in each cluster divided by experimental group: *App*^*WT*^, *App*^*NL-G-F*^ at 3 months and *App*^*NL-F-G*^ at 6 months (normalized expression scaled by gene is shown). **f**, Representative images of human microglia engrafted in the brain of *App*^*NL-G-F*^ mice at 6 months of age and labeled with human-specific CD9 (DAM) and HLA-DR/DQ (HLA microglia) antibodies. Scale bars, 25 μm (upper panel) and 50 μm (lower panel). APP–NLGF_3m, *App*^*NL*-*G*-*F*^ 3 months; APP–NLGF_6m, *App*^*NL*-*G*-*F*^ 6 months; APP–WT_6m, *App*^*WT*^ 6 months.

**Extended Data Fig. 4:**
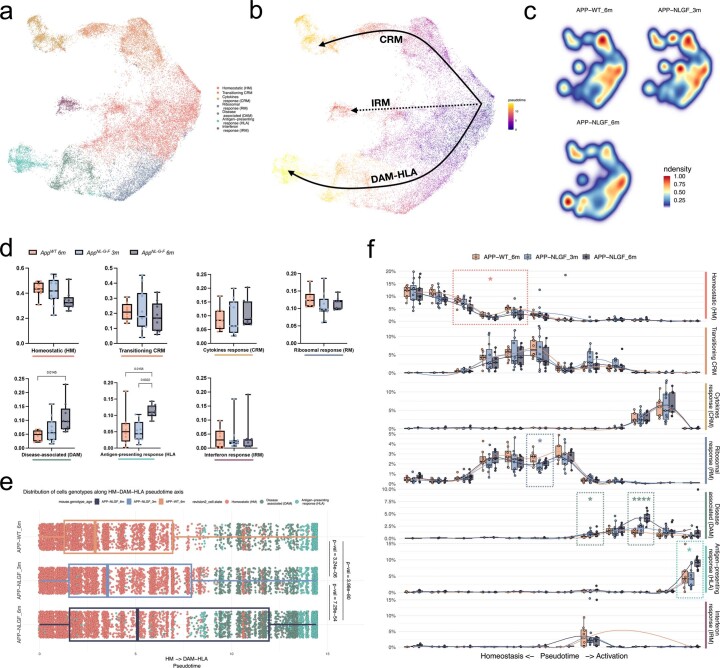
Extended pseudotime analysis, including transplanted microglia isolated from 3 months old *App*^*NL-G-F*^ mice. **a**, UMAP plot of the 38,360 mouse microglia isolated from 3 and 6 months *App*^*NL-G-F*^ and *App*^*WT*^ mice passing quality control, coloured by annotated cell states and after removal of CAMs, other myeloid, low quality and proliferating clusters. **b**, Phenotypic trajectory followed by the human microglia after exposure to amyloid-β *in vivo*, obtained by an unbiased pseudotime ordering with Monocle 3. **c**, Density plots displaying the average distribution of human microglia transplanted in 3 months old *App*^*NL-G-F*^ (n = 11) 6 months old *App*^*NL-G-F*^ (n = 9) and *App*^*WT*^ (n = 8) mice (density is normalized to sample size). **d**, Box plot showing proportion of cells across all identified clusters (eEach dot represents a single mouse, n = 8 for *App*^*WT*^, n = 11 for *App*^*NL-G-F*^ 3 m, n = 9 for *App*^*NL-G-F*^ 6 m). **e**, Distribution of cells from different host mouse genetic backgrounds and ages (y-axis) across the DAM and HLA transcriptional trajectories colored by clusters shown in **a** (Each dot represents a single cell, n = 8 mice (8,974 cells) for *App*^*WT*^, n = 11 mice (10,822 cells) for *App*^*NL-G-F*^ 3 m, n = 9 mice (12,197 cells) for *App*^*NL-G-F*^ 6 m). **f**, Phenotypic trajectory followed by the human microglia obtained by an unbiased pseudotime ordering with *Monocle 3*. Proportion of cells (y-axis) present in each cell state as in **a** (rows) at different stages of the binned pseudotime trajectory (x-axis) in 3 and 6 months old *App*^*NL-G-F*^ and *App*^*WT*^ mice (Each dot represents a single mouse, n = 8 for *App*^*WT*^, n = 11 for *App*^*NL-G-F*^ 3 m, n = 9 for *App*^*NL-G-F*^ 6 m; *p < 0.05, ****p < 0.001). Box plots in **d, e** and **f** are limited by lower and upper quartiles and midline indicates median; whiskers show minimum to maximum value (**d**) or extend from the box to the smallest or largest value no further than 1.5*inter-quartile range (**e** and **f**). One-way ANOVA with Tukey’s multiple comparisons as post-hoc test, alpha = 0.05, significance was set as P-adjusted value < 0.05.

**Extended Data Fig. 5:**
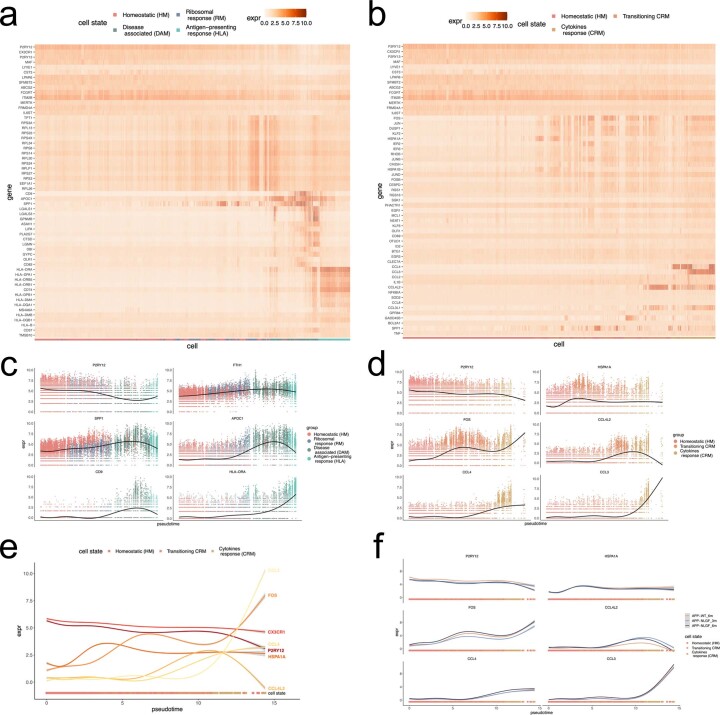
Extended pseudotime analysis, including transplanted microglia isolated from 3 months old *App*^*NL-G-F*^ mice (continuation). **a, b**, Heatmaps displaying the cells ordered by the (**a**) HM-DAM-HLA and (**b**) HM-CRM pseudotime axis, coloured by single-cell levels of expression of the top markers per cluster. **c, d**, Expression profile of selected genes across the (**c**) HM-DAM-HLA and (**d**) CRM pseudotime axis averaging all experimental groups in the dataset. **e**, Comparison of the expression profile of selected CRM markers across the trajectory. **f**, Expression profile of selected genes across the CRM pseudotime axis as in **d** divided into the different experimental groups: *App*^*WT*^, *App*^*NL-G-F*^ 3 M and *App*^*NL-F-G*^ 6 M.

Trajectory analysis indicated that microglia follow three main activation routes from homeostatic states toward four distinct transcriptional cell states: DAM, HLA, CRM and IRM (Fig. 3a,b and Extended Data Figs. 4 and 5). Whereas the DAM to HLA and IRM trajectories partially overlap, CRM deflects early during the phenotypic transition, resulting in an apparently independent response program (Fig. 3a,b and Extended Data Figs. 4 and 5). HLA appears as a continuation of the DAM response, resulting in a DAM to HLA trajectory (Fig. 3a,b and Extended Data Fig. 5). To confirm the trajectory inference, we performed a time course analysis using one of the cell lines (BIONi-010C) to analyze the transcriptomic changes of microglia isolated from 3- (onset of plaque deposition) and 6-month-old *App*^*NL-G-F*^ mice (Fig. 3c,d and Extended Data Fig. 4). We confirm the same three independent trajectories (Extended Data Fig. 4a–c), with an age-dependent increase in DAM and HLA clusters, correlating with amyloid deposition (Extended Data Fig. 4d–f). The changes over time are not simple shifts in cell numbers across the transcriptional profiles, because overall levels of expression of homeostatic markers within the HM cluster specifically decreased (WT > 3 months > 6 months), and disease-associated and antigen-presenting markers within DAM and HLA clusters specifically increased (WT < 3 months < 6 month) over the disease course (Fig. 3e). In addition, we performed costaining of DAM (CD9) and HLA (HLA-DR/DQ) and observed that whereas all HLA-positive cells showed CD9 expression, not all CD9-positive cells showed HLA signal, thus supporting the prediction of a phenotypic continuum between DAM and HLA (Fig. 3f).

Interestingly, both the presence and magnitude of HLA and CRM phenotypic programs appear to be human-specific features, as these clusters have not been reported previously in mouse models even in advanced stages of disease^3–5,9^. To confirm this further, we compared the transcriptional profile of engrafted human microglia with mouse cells isolated from 6-month-old *Rag2*^−/−^ *Il2rγ*^−/−^ *hCSF1*^*KI*^ *App*^*NL-G-F*^ (*n* = 1,024 cells, 2 mice) and with a dataset of immunocompetent *App*^*NL-G-F*^ and WT mice^3^ that we published previously (*n* = 2,779 cells, 4 mice). We integrated these two datasets, performed clustering analysis and investigated whether the HLA and CRM transcriptomic signatures were present. A small number of cells were consistent with HLA and CRM profiles, but, unlike human microglia, they did not seem to increase in response to Aβ pathology (Extended Data Fig. 3a,b). This low percentage of MHC-II-expressing cells is consistent with previous reports^31^.

We also explored whether the relationship between DAM and HLA could be the equivalent to that of DAM1 versus DAM2 observed in mice^4^. Direct comparison between these responses confirmed that the HLA response is unique to human microglia, and different from the murine DAM2 (Extended Data Fig. 3c). Both HLA and DAM2 showed increased expression of DAM genes (such as *CD9*, *CSTD* and *GPNMB*) compared with homeostatic cells, but these slightly decrease in the DAM–HLA human transition while they keep increasing in the mouse DAM1–DAM2 trajectory. Moreover, HLA is characterized by a specific induction of antigen-presenting molecules (including *HLA-DQB1*, *HLA-DRB5* and *CD74*) and a smaller enrichment in ribosomal genes (such as *RPS3*, *RPS9* and *RPS19*) which are instead downregulated in DAM2. This indicates that human microglia display an elaborate response to Aβ pathology. The HLA response suggests that they engage in antigen presentation of phagocyted material (for example, Aβ). Although the potential interaction of HLA microglia and T cells cannot be assessed in the chimeric model system, our findings emphasize the need of further research efforts in that direction. Overall human microglia display a complex, multipronged response to Aβ pathology, including some programs that share features of the previously described DAM, IRM and TRM responses^3,4^, but also two human-specific CRM and HLA transcriptional states.

### *TREM2* and *APOE* control microglial cell-state transition

**Fig. 4:**
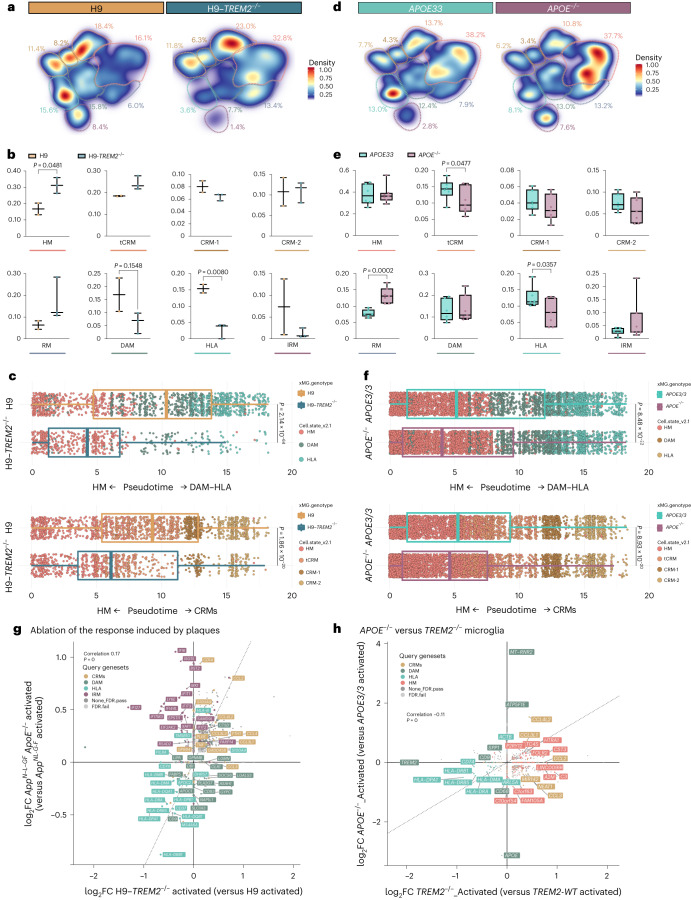
TREM2 and APOE differentially modulate the transition to DAM and HLA states. **a**,**d**, Density plots displaying the average distribution of human H9-*WT* (*n* = 2) and H9-*TREM2*^−/−^ (*n* = 3) (**a**), and human *APOE3/3* (*n* = 6) and *APOE*^−/−^ (*n* = 7) (**d**) microglia transplanted in *App*^*NL-G-F*^ mice. Density is normalized by sample size. **b**,**e**, Distribution and proportion of cells across all identified clusters for H9-*WT* (*n* = 2) and H9-*TREM2*^−/−^ (*n* = 3) (**b**), and *APOE3/3* (*n* = 6) and *APOE*^−/−^ (*n* = 7) (**e**) transplanted microglia. Dots represent single mice. **c**,**f**, Phenotypic trajectory followed by H9-*WT* (*n* = 2 mice and 3,282 cells) and H9-*TREM2*^−/−^ (*n* = 3 mice and 1,301 cells) (**c**), and *APOE3/3* (*n* = 6 mice and 7,894 cells) and *APOE*^−/−^ (*n* = 7 mice and 14,012 cells) (**f**) human microglia obtained by an unbiased pseudotime ordering with Monocle 3. Proportion of cells (*y* axis) at different stages of the pseudotime trajectory (*x* axis), colored as shown in Fig. 1a. Dots represent single cells. **g**, Correlation analysis of the logFC in microglia transplanted in *App*^*NL-G-F*^ *ApoE*^−/−^ versus *App*^*NL-G-F*^ mice (*y* axis from Fig. 4) and H9-*TREM2*^−/−^ versus H9-*WT* from *App*^*NL-G-F*^ mice (*x* axis) (Pearson’s correlation, *R* = 0.17, differentially expressed genes adjusted using Bonferroni correction and colored as in Fig. 1b; ‘activated’ indicates that differential expression was performed comparing cell states reactive to pathology, excluding homeostatic or transitioning clusters). **h**, Correlation analysis of the logFC in *TREM2*^−/−^ (*x* axis) and *APOE*^−/−^ (*y* axis) microglia transplanted in *App*^*NL-G-F*^ mice (Pearson’s correlation, *R* = −0.11, differentially expressed genes adjusted using Bonferroni correction and colored as in Fig. 1b; ‘activated’ indicates that differential expression was performed comparing reactive cell states, excluding homeostatic or transitioning clusters). Box plots in **b**,**c**,**e**,**f** are limited by lower and upper quartiles and midline indicates median; whiskers show minimum to maximum value (**b**,**e**) or extend from the box to the smallest or largest value no further than 1.5× interquartile range (**c**,**f**). Unpaired *t*-test with Welch’s correction, two-tailed, alpha = 0.05, significance was set as *P* < 0.05.

**Extended Data Fig. 6:**
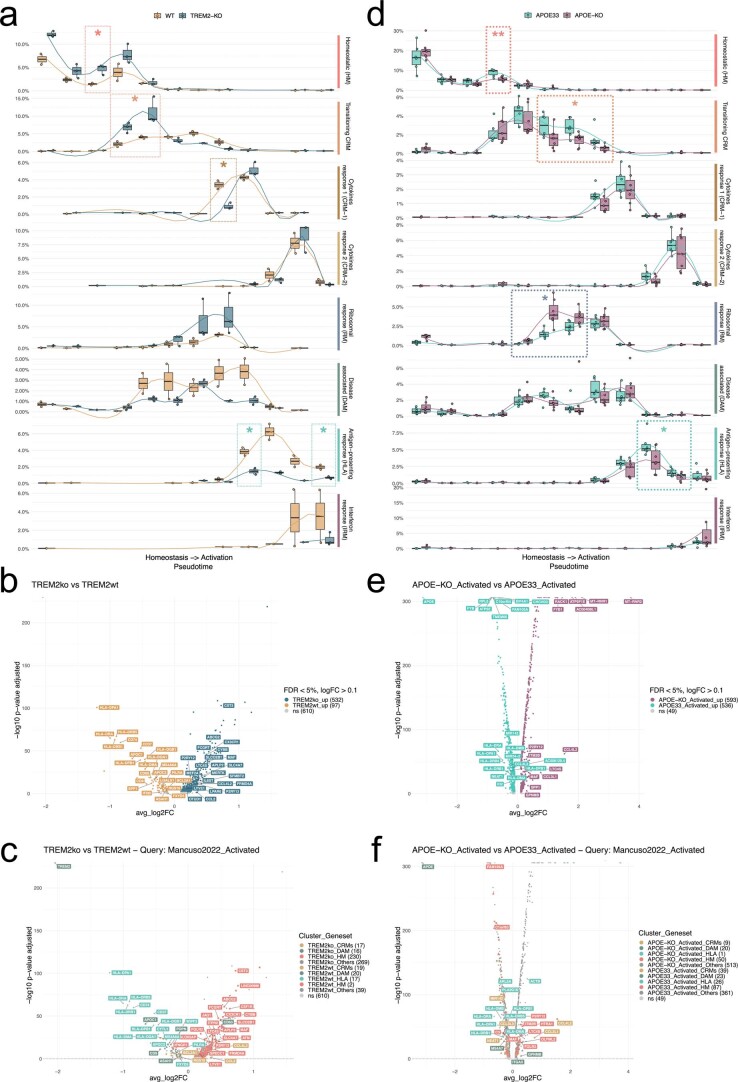
Extended analysis on the differential impact of TREM2 and APOE deficiency in the transplanted microglia. **a**, Phenotypic trajectory followed by TREM2 deficient and control microglia, obtained by an unbiased pseudotime ordering with *Monocle 3*. Proportion of cells (y-axis) present in each cell state as in Fig. 1b (rows) at different stages of the binned pseudotime trajectory (x-axis), colored by host genotypes (each dot represents a single mouse, n = 2 mice H9-*WT*, n = 3 mice H9-*TREM2*^−/−^). **b, c**, Volcano plots showing a paired comparison between *TREM2*^−/−^ and WT microglia transplanted in *App*^*NL-G-F*^, coloured by **(b)** cells genotype and **(c)** cluster as in Fig. 1b. The number of significant genes per condition is reported in brackets in the legend (Log2 Fold-Change threshold > 0.1). Adjusted p-value threshold < 0.05 (Wilcoxon rank-sum test, P-values adjusted with Bonferroni correction based on the total number of genes in the dataset, ns = not significant). **d**, Phenotypic trajectory followed by *APOE* deficient microglia compared to isogenic *APOE3/3* controls, obtained by an unbiased pseudotime ordering with *Monocle 3*. Proportion of cells (y-axis) present in each cell state as in Fig. 1b (rows) at different stages of the binned pseudotime trajectory (x-axis), colored by host genotypes (each dot represents a single mouse, n = 6 *APOE3/3* and n = 7 for *APOE*^−/−^). **e, f**, Volcano plots showing a paired comparison between *APOE*^−/−^ and *APOE3/3* microglia transplanted in *App*^*NL-G-F*^, coloured by **(e)** cells genotype and **(f)** cluster as in Fig. 1b. The number of significant genes per condition is reported in brackets in the legend (Log2 Fold-Change threshold > 0.1). Adjusted p-value threshold < 0.05 (Wilcoxon rank-sum test, P-values adjusted with Bonferroni correction based on the total number of genes in the dataset, ns = not significant). Box plots in **a** and **d**, are limited by lower and upper quartiles and midline indicates median; whiskers extend from the box to the smallest or largest value no further than 1.5*inter-quartile range. Unpaired *t*-test with Welch’s correction, two-tailed, alpha = 0.05, significance was set as P-value < 0.05, *p < 0.05, **p < 0.01.

We investigated the molecular mechanisms governing the transition toward different transcriptional cells states in human microglia in vivo. TREM2 is one of the main cell-autonomous mediators of the microglial response to Aβ plaques in mice^4,5,32^, and thus we wondered whether it was able to modify human microglial cell states in a similar way. We analyzed 3,282 H9 (*n* = 2) and 1,301 H9-*TREM2*^−/−^ (*n* = 3) microglia from 6-month-old *App*^*NL-G-F*^ mice and observed a profound suppression of both DAM and HLA responses, but no significant alterations in the CRM profile (Fig. 4a–c and Extended Data Fig. 6a–c). These data confirm that TREM2 is necessary for the DAM and HLA responses in human microglia, confirming and extending a previous study showing that *TREM2*-deficient human microglia have an impaired DAM phenotype^33^.

ApoE is critical for the transition to DAM in mouse models^5^. Therefore, we transplanted and analyzed the transcriptomes of 7,894 *APOE3/3* (*n* = 6) and 11,799 *APOE*^−/−^ (*n* = 7) human microglia isolated from 6-month-old *App*^*NL-G-F*^ mice. We observed that the ablation of *APOE* had mild effects on the microglial cell states, with no impact on DAM or CRM profiles, but caused a significant reduction in HLA microglia (Fig. 4d–f and Extended Data Fig. 6d–f). This suggests that although microglial APOE may not be necessary to initiate the response to Aβ plaques, it is required for the transition to the HLA state.

Our data show that TREM2 is a powerful inductor of the phenotypic transition to both DAM and HLA states in human microglia and place APOE as a subsequent factor needed for the shift from DAM to HLA, highlighting species-specific differences in how microglia react against the Aβ present in plaques.

### Different activation programs against distinct forms of Aβ

**Fig. 5:**
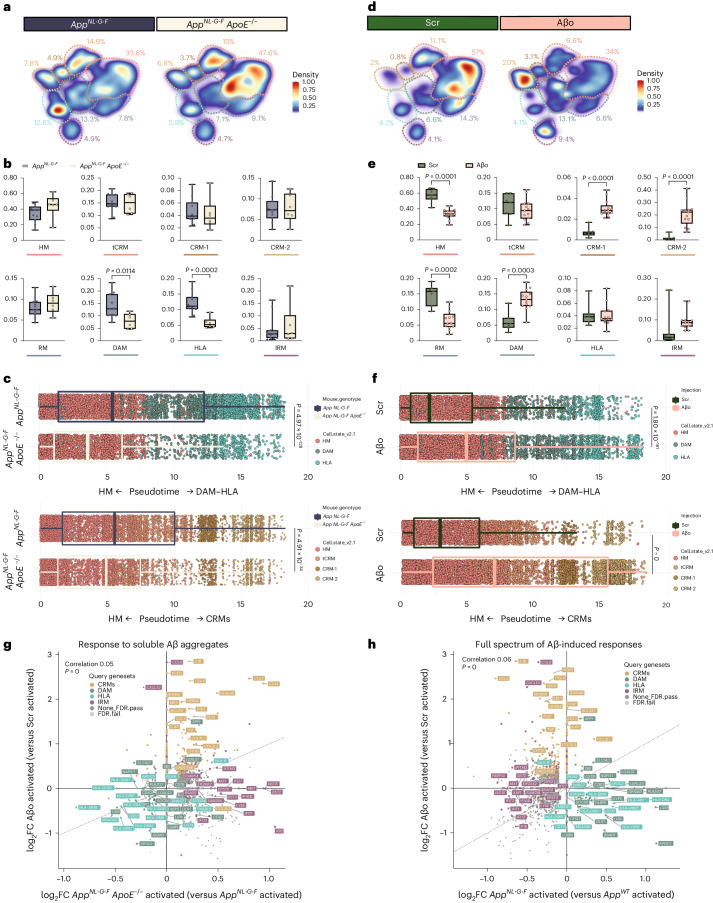
Human microglia display a differential response to Aβ plaques and Aβo. **a**,**d**, Density plots displaying the average distribution of human microglia transplanted in *App*^*NL-G-F*^ (*n* = 11) and *App*^*NL-G-F*^ *ApoE*^−/−^ (*n* = 6) mice (**a**), and human microglia transplanted in *App*^*WT*^ mice and challenged with scrambled peptide (Scr, *n* = 7) or Aβo (*n* = 13) (**d**). Density is normalized by sample size. **b**,**e**, Distribution and proportion of cells across all identified clusters for microglia transplanted in *App*^*NL-G-F*^ (*n* = 11) and *App*^*NL-G-F*^ *ApoE*^−/−^ (*n* = 6) mice (**b**), and Scr/Aβo injected mice (**e**). Dots represent single mice. **c**,**f**, Phenotypic trajectory followed by human microglia transplanted in *App*^*NL-G-F*^ (*n* = 11 and 14,649 cells) and *App*^*NL-G-F*^ *ApoE*^−/−^ (*n* = 6 and 7,738 cells) mice (**c**), and challenged with scrambled peptide (Scr, *n* = 7 and 10,967 cells) or Aβo (*n* = 13 and 18,691 cells) (**f**), obtained by an unbiased pseudotime ordering with Monocle 3. Proportion of cells from different mouse hosts (*y* axis) at different stages of the pseudotime trajectory (*x* axis), colored as shown in Fig. 1a. Dots represent single cells. **g**, Correlation analysis of the logFC in microglia either challenged with Aβο versus Src (*y* axis) or transplanted in *App*^*NL-G-F*^ *ApoE*^−/−^ versus *App*^*NL-G-F*^ mice (*x* axis) (Pearson’s correlation, *R* = 0.05, differentially expressed genes were adjusted using Bonferroni correction and colored according to clusters in Fig. 1a). **h**, Correlation analysis of the logFC in microglia either challenged with Aβ soluble aggregates (Aβο) versus scrambled peptide (Src, *y* axis) or transplanted in *App*^*NL-G-F*^ mice (*x* axis) (Pearson’s correlation, *R* = 0.06, differentially expressed genes adjusted using Bonferroni correction). Box plots in **b**,**c**,**e**,**f** are limited by lower and upper quartiles and midline indicates median; whiskers show minimum to maximum values (**b**,**e**) or extend from the box to the smallest or largest value no further than 1.5× interquartile range (**c**,**f**). Unpaired *t*-test with Welch’s correction, two-tailed, alpha = 0.05, significance was set as *P* < 0.05. Scr, scrambled peptide.

**Extended Data Fig. 7:**
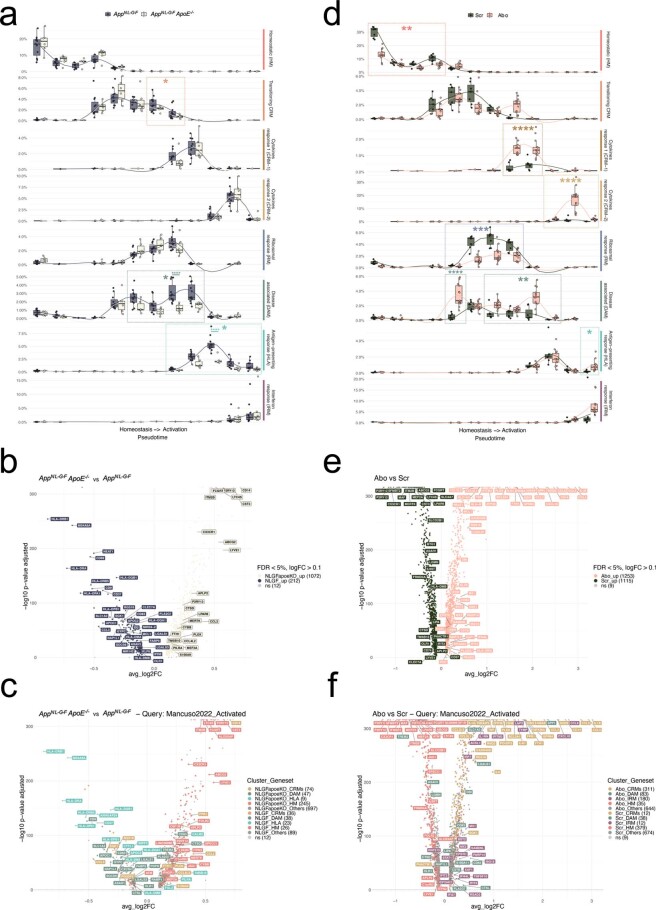
Extended analysis on the differential effects of amyloid-β plaques and soluble aggregates on microglia cell states. **a**, Phenotypic trajectory followed by the human microglia transplanted in *App*^*NL-G-F*^ *ApoE*^−/−^ and *App*^*NL-G-F*^, obtained by an unbiased pseudotime ordering with *Monocle 3*. Proportion of cells (y-axis) present in each cell state as in Fig. 1b (rows) at different stages of the binned pseudotime trajectory (x-axis), colored by host genotypes (dots represent single mice, n = 11 *App*^*NL-G-F*^, n = 6 *App*^*NL-G-F*^ *ApoE*^−/−^). **b, c**, Volcano plots showing a paired comparison between microglia transplanted in *App*^*NL-G-F*^ *ApoE*^−/−^ and *App*^*NL-G-F*^ mice, coloured by **(b)** host genotype and **(c)** cluster as in Fig. 1a. The number of significant genes per condition is reported in brackets in the legend (Log2 Fold-Change threshold > 0.1). Adjusted p-value threshold < 0.05 (Wilcoxon rank-sum test, P-values adjusted with Bonferroni correction based on the total number of genes in the dataset, ns = not significant). **d**, Phenotypic trajectory followed by the human microglia after injection of amyloid-b oligomers (Aβο) or scrambled peptide (Scr), obtained by an unbiased pseudotime ordering with *Monocle 3*. Proportion of cells (y-axis) present in each cell state as in Fig. 1a (rows) at different stages of the binned pseudotime trajectory (x-axis), colored by cells genotypes (dots represent single mice, n = 7 for Scr, n = 13 Aβο). **e, f**, Volcano plots showing a paired comparison between microglia transplanted in *App*^*WT*^ mice and treated with Aβο or Scr, coloured by **(e)** treatment and **(f)** cluster as in Fig. 1a. The number of significant genes per condition is reported in brackets in the legend (Log2 Fold-Change threshold > 0.1). Adjusted p-value threshold < 0.05 (Wilcoxon rank-sum test, P-values adjusted with Bonferroni correction based on the total number of genes in the dataset, ns = not significant). Box plots in **a** and **d**, are limited by lower and upper quartiles and midline indicates median; whiskers extend from the box to the smallest or largest value no further than 1.5*inter-quartile range. Unpaired *t*-test with Welch’s correction, two-tailed, alpha = 0.05, significance was set as P-value < 0.05, *p < 0.05, **p < 0.01, ***p < 0.001,****p < 0.0001.

We wondered whether the distinct transcriptional trajectories adopted by human microglia might be caused by different forms of Aβ pathology. We used a genetic approach to reduce the Aβ plaques in the mouse brain by knocking out *ApoE* (*App*^*NL-G-F*^ *ApoE*^−/−^). Note that in these experiments, the endogenous mouse *ApoE* gene is inactivated, as opposed to the experiments described above where only the *APOE* gene in the microglia was deleted. As previously reported, this leads to a significant reduction of Aβ plaques^34^, as well as a diminished plaque size (Supplementary Fig. 5b–e). We analyzed 22,387 microglia isolated from 6-month-old *App*^*NL-G-F*^ (*n* = 11) and *App*^*NL-G-F*^ *ApoE*^−/−^ mice (*n* = 6, 2 independent induced pluripotent stem cells (iPSC) lines per group) (Supplementary Figs. 2 and 3). Clustering analysis revealed a significant reduction in the recruitment of microglia into DAM and HLA phenotypes, with no alterations in the CRM response (Fig. 5a,b and Extended Data Fig. 7). Trajectory analysis confirmed a strong reduction in the proportion of cells transitioning into the DAM–HLA transcriptional axis (Fig. 5c and Extended Data Fig. 7). These observations indicate that there is a non-cell-autonomous effect of ApoE secreted, for instance, from astroglia on microglia. This effect could be indirect via the modulation of Aβ aggregation or Aβ fibril structure or by modulating other cells or cellular processes.

To further assess whether the remaining CRM response is mediated by soluble Aβo, we injected recombinant soluble Aβo (5 μl at 10 μM, *n* = 13) or scrambled peptide (Scr, *n* = 7, 2 independent cell lines per group) in the ventricle of xenotransplanted ~3-month-old *App*^*WT*^ mice^14^. We isolated and sequenced 29,658 microglia 6 h after injection (Supplementary Fig. 2). Whereas microglia exposed to Scr peptides remain largely homeostatic, microglia challenged with soluble Aβo adopt a CRM transcriptional state (Fig. 5d,e and Extended Data Fig. 7). Contrary to Aβ plaques, and despite that these soluble Aβo partially shifted cells into a DAM state, they did not induce an HLA response. Trajectory analysis confirmed that soluble Aβo-treated cells undergo an almost complete phenotypic transition across CRM-1 and CRM-2 clusters (Fig. 5f). CRM-2 is at the extreme of this trajectory and the dominant cell state after acute injection of high levels of Aβo (Fig. 5f and Extended Data Fig. 7), consistent with our previous observations^14^. Several variables can affect the response to these aggregates, including concentration of Aβ, levels of aggregation or traces of bacterial endotoxin from the manufacturing process. We analyzed independent batches of soluble Aβ aggregates and could exclude that these sources of variation affected the induction of CRM. Thus, overall the microglial response consists of independent cell states that coexist in the *App*^*N-L-GF*^ model for AD and are elicited by Aβ plaques (DAM, HLA) and soluble Aβo species (CRM), respectively (Fig. 5g,h).

### The response of engrafted microglia mimics the human brain

**Fig. 6:**
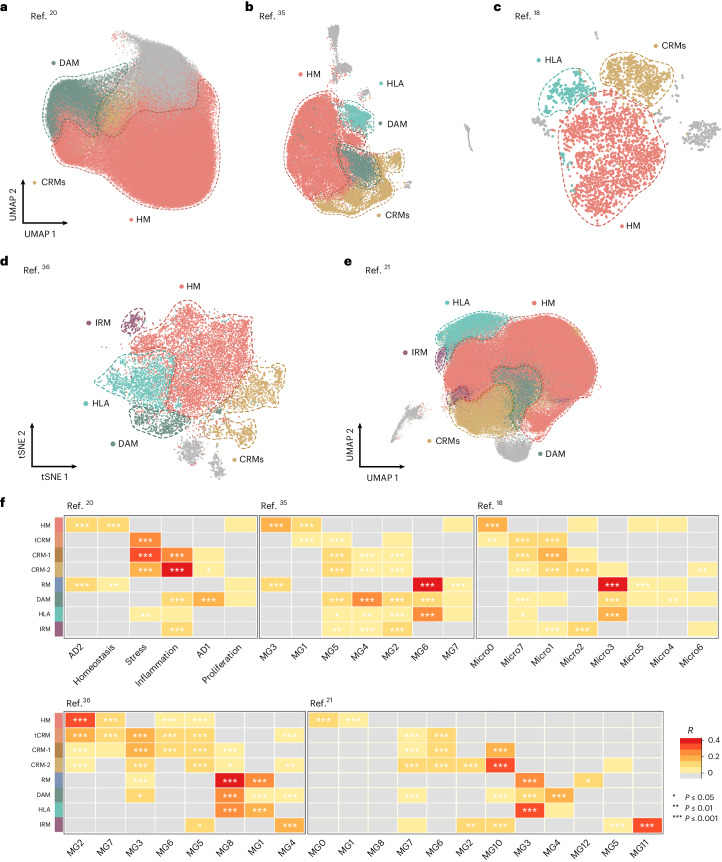
Single-microglial nuclei from human postmortem brain. **a**–**e**, Human snRNA-seq datasets from Gerrits et al.^20^ (*n* = 176,136) (**a**), Sayed et al.^35^ (*n* = 28,767) (**b**), Zhou et al.^18^ (*n* = 3,978) (**c**), Olah et al.^36^ (*n* = 16,242) (**d**) and Sun et al.^21^ (*n* = 194,000) (**e**) re-analyzed to reproduce their original embeddings and annotated with our xenotransplanted microglial profiles as in Fig. 1b. **f**, Pairwise Pearson correlation between logFC of all differentially expressed genes (logFC cut-off set at 0.25, *P* < 0.05) of each microglial subtype and logFC of all differentially expressed genes (*P* < 0.05) of clusters from each human snRNA-seq study, with significance set at a *P*-adjusted value < 0.05 (*P ≤ 0.05, **P ≤ 0.01, ***P ≤ 0.001). Only positive correlations are depicted here (*r* > 0). Additional correlations are shown in Extended Data Figs. 6 and 7. scRNA-seq, single cell RNA-sequencing.

**Extended Data Fig. 8:**
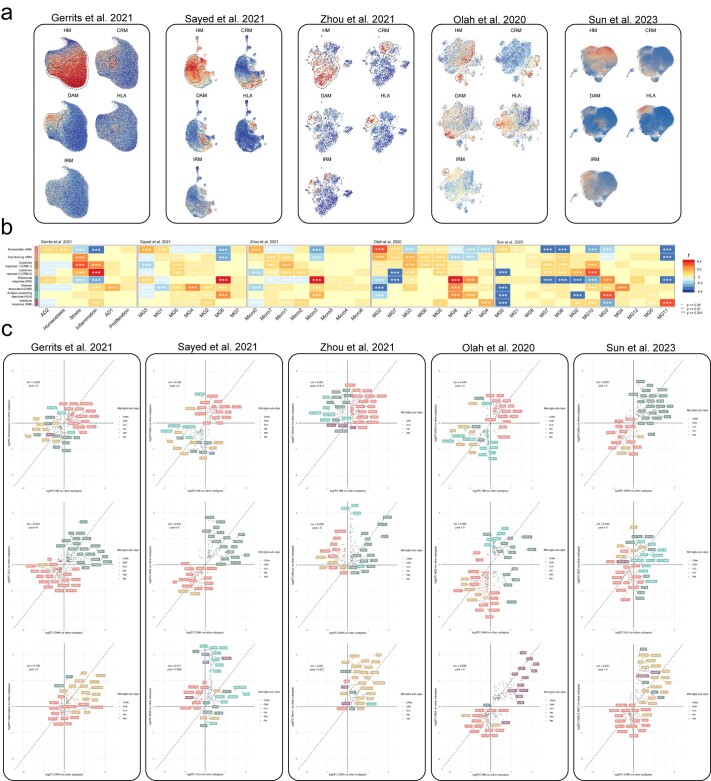
Extended analysis of the single nuclei from human post-mortem brains (continuation). **a**, UMAP plots as in Fig. 6, coloured by the combined level of expression of groups of genes that characterise distinct microglial transcriptional states from xenotransplanted microglia. **b**, Full set of pairwise Pearson correlations between logFC of all DE genes (logFC cut off set at 0.25, P-value < 0.05) of each microglia sub-type and logFC of all DE genes (p < 0.05) of clusters from each human snRNA-seq study, with significance set at a P-adjusted value < 0.05 (*p < =0.05, **p < =0.01, ***p < =0.001). **c**, Representative scatter plots showing the correlation analysis of the logFC between xenotransplanted and single nuclei post-mortem microglia. The labels are coloured by cell state as in Fig. 1.

We wondered whether the two main modalities of human microglial response to Aβ pathology are preserved in AD human brain. We extracted microglial single-nuclei RNA sequencing data from the four most recent independent single-nuclei sequencing studies investigating the transcriptome of human microglia in the AD brain, providing 176,136 nuclei from Gerrits et al.^20^, 28,767 from Sayed et al.^35^, 3,978 from Zhou et al.^18^ and 194,000 from Sun et al.^21^, respectively. A fifth study from Olah et al.^36^ provided 16,242 single-cell transcriptomes from freshly isolated microglia from surgical resection. We reproduced their original clustering analysis (Supplementary Fig. 6 and Extended Data Fig. 8) and re-analyzed these data using the transcriptomic profiles from transplanted microglia (Fig. 6). Despite discrepancies in the numbers and proportions of clusters reported across these studies, we were able to identify populations of nuclei weakly (*R* = 0.2–0.4) to moderately (*R* = 0.4–0.6) or strongly (*R* > 0.6) correlating to all the microglial cell states, especially HM, DAM, HLA and CRM (Fig. 6 and Extended Data Fig. 8), as well as a clear IRM signature in the fourth datasets from single cells (Fig. 6). Of note, none of the postmortem datasets alone covered the full phenotypic diversity in response to Aβ as observed in the xenotransplanted microglia (Fig. 3d). For example, in Gerrits et al.^20^ DAM corresponds to *AD1*, and CRM to *Inflammation*, but the HLA signature is absent (Extended Data Fig. 3d and Fig. 6a,d). In Zhou et al.^18^, all signatures are present, but they converge in a limited number of clusters (*Micro1*, *Micro4* and *Micro3*). In Sayed et al.^35^, although the main Aβ-induced signatures are present, DAM and HLA, and IRM and CRM show partial overlap (Fig. 6b,d). For Sun et al.^21^, we were able to correlate our HM (MG0 and MG1), DAM (MG3, MG4 and MG10), HLA (MG4) and CRM (MG6 and MG7) states. Certain transcriptomic states from primary samples that were correlated with tau pathology (for example, *AD2*)^20^ overlap with our HM signature. In Olah et al.^36^, we also observed an overlap between the transcriptional states of transplanted microglia and those from human biopsies^36^, consistent with our HM (*MG2*), DAM (*MG1*), HLA (*MG8*), CRM (*MG3*) and IRM (*MG4*) clusters, respectively (Extended Data Fig. 3d and Fig. 6d,e). Microglial states resembling HLA and CRM were shown before in brains from AD subjects by Lau et al.^37^. Additional cell states described in some of these postmortem studies are not captured in our xenotransplantation model, which might reflect the response to additional pathologies in the late phase of AD or the heterogeneity of pathology in old age (Fig. 6d and Extended Data Fig. 8). Overall, it appears that the cell states in transplanted microglia are present in the human AD brain and that the data generated here in the well-controlled xenograft system are useful to understand the response of human microglia to amyloid pathology in patients.

### AD risk genes modulate microglial cell states in vivo

**Fig. 7:**
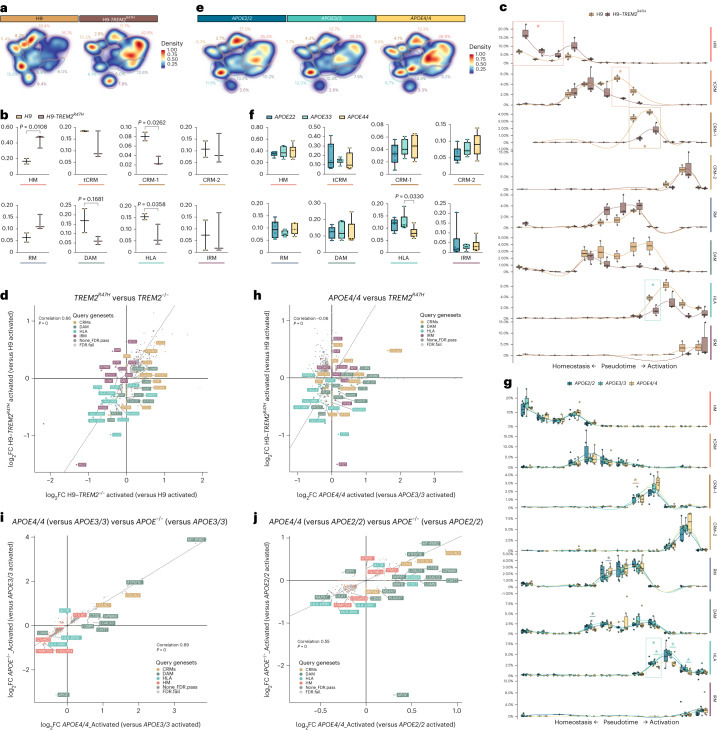
AD genetic risk modifies the response of human microglia to Aβ pathology. **a**,**e**, Density plots displaying the average distribution of human H9-*WT* (*n* = 2) and H9-*TREM2*^*R47H*^ (*n* = 3) (**a**), and *APOE2/2* (*n* = 6), *APOE3/3* (*n* = 6) and *APO4/4* (*n* = 6) human microglia (**e**) transplanted in *App*^*NL-G-F*^ mice. **b**,**f**, Distribution and percentage of cells across all identified clusters for H9-*WT* (*n* = 2) and H9-*TREM2*^*R47H*^ (*n* = 3) (**b**), and *APOE2/2* (*n* = 6), *APOE3/3* (*n* = 6) and *APO4/4* (*n* = 6) (**f**). Dots represent single mice. **c**,**g**, Phenotypic trajectory followed by H9-*WT* (*n* = 2) and H9-*TREM2*^*R47H*^ (*n* = 3) (**c**), and *APOE2/2* (*n* = 6), *APOE3/3* (*n* = 6) and *APO4/4* (*n* = 6) (**g**) human microglia obtained by an unbiased pseudotime ordering with Monocle 3. Proportion of cells (*y* axis) over the binned pseudotime trajectory (*x* axis), colored by genotypes shown in **a**. Dots represent single mice (*P < 0.05). **d**,**h**, Correlation of the logFC in *TREM2*^*R47H*^ versus H9-*WT* (*y* axis) and H9-*TREM2*^−/−^ versus H9-*WT* (*x* axis) (**d**), and *TREM2*^*R47H*^ versus H9-*WT* (*y* axis) and *APOE4/4* versus *APOE3/3* (*x* axis) (**h**) microglia transplanted in *App*^*NL-G-F*^ mice (Pearson’s correlation, *R* = 0.17, differentially expressed genes were adjusted using Bonferroni correction and colored as in Fig. 1b). **i**,**j**, Comparison of transcriptomic profiles of *APOE4/4* (versus *APOE3/3*) versus *APOE*^−/−^ (versus *APOE3/3*) (**i**), and APOE4/4 (versus *APOE2/2*) versus *APOE*^−/−^ (versus *APOE2/2*) (**j**) microglia transplanted in *App*^*NL-G-F*^ mice (Pearson’s correlation, differentially expressed genes were adjusted using Bonferroni correction and colored according to clusters in Fig. 1b). Box plots in **b**,**c**,**f**,**g** are limited by lower and upper quartiles and midline indicates median; whiskers show minimum to maximum values (**b**,**f**) or extend from the box to the smallest or largest value no further than 1.5 × interquartile range (**c**,**g**). Unpaired *t*-test with Welch’s correction, two-tailed, alpha = 0.05, significance was set as *P* < 0.05 (**b**,**c**); or one-way ANOVA with Tukey’s multiple comparisons as post hoc test, alpha = 0.05, significance was set as *P*-adjusted value < 0.05 (**f**,**g**). In **d**,**h**,**i**, ‘activated’ indicates that differential expression was performed comparing reactive cell states, excluding homeostatic or transitioning clusters.

**Extended Data Fig. 9:**
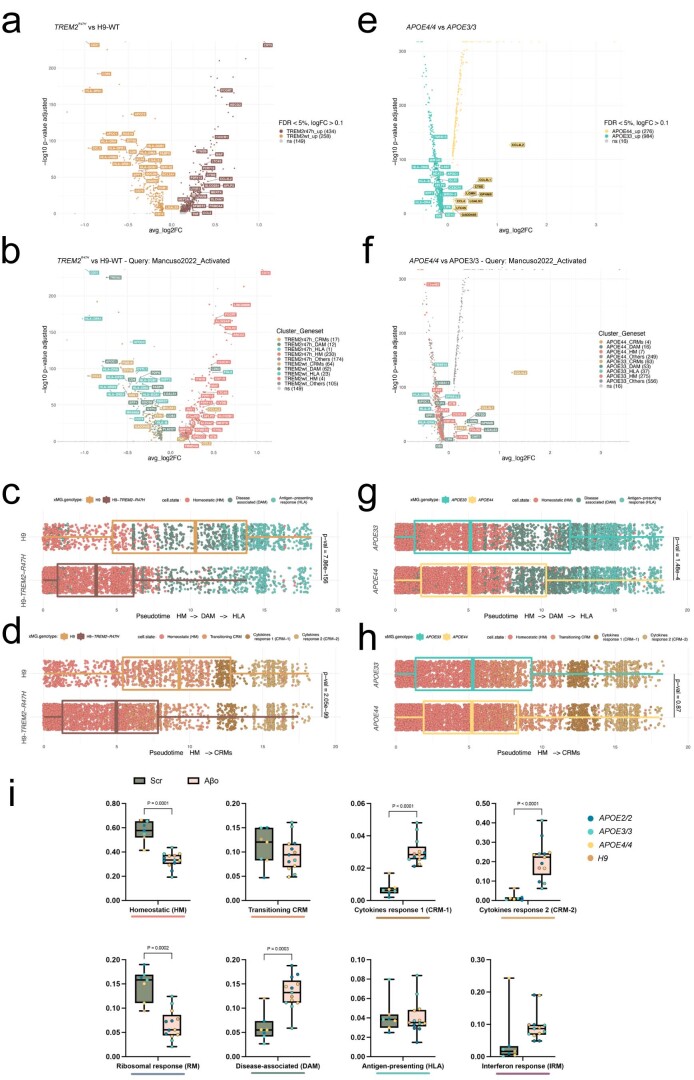
Extended analysis of the impact of clinical mutations on microglial cell states. **a, b**, Volcano plots showing a paired comparison between *TREM2*^*R47H*^ and *TREM2-WT* microglia transplanted in *App*^*NL-G-F*^ mice and coloured by **(a)** cells genotype or **(b)** cluster as in Fig. 1a. The number of significant genes per condition is reported in brackets in the legend (Log2 Fold-Change threshold > 0.1). Adjusted p-value threshold < 0.05 (Wilcoxon rank-sum test, P-values adjusted with Bonferroni correction based on the total number of genes in the dataset, ns = not significant). **c, d**, Distribution of *TREM2*^*R47H*^ and *TREM2-WT* cells across **(c)** DAM-HLA and **(d)** CRM transcriptional trajectories. Note the shift in transcriptional states in both the DAM-HLA and CRM axes (each dot represents a single cell, n = 2 (3,282 cells) H9-*WT*, n = 3 (5,845 cells) H9-*TREM2*^*R47H*^). **e, f**, Volcano plots showing a paired comparison between *APOE4/4* and *APOE3/3* microglia transplanted in *App*^*NL-G-F*^ mice and coloured by **(e)** cells genotype or **(f)** cluster as in Fig. 1a. The number of significant genes per condition is reported in brackets in the legend (Log2 Fold-Change threshold > 0.1). Adjusted p-value threshold < 0.05 (Wilcoxon rank-sum test, P-values adjusted with Bonferroni correction based on the total number of genes in the dataset, ns = not significant). **g, h**, Distribution of *APOE4* and *APOE3* cells across **(g)** DAM and HLA and **(h)** CRM transcriptional trajectories (each dot represents a single mouse, n = 6 (7,894 cells) for *APOE3/3*, n = 6 (10,151 cells) for *APO4/4*). **i**, Distribution and proportion of cells across all identified clusters as in Fig. 5e, coloured by the microglial APOE genotype (each dot represents a single mouse, n = 7 for Scr, n = 13 Aβο). Box plots in **c, d, g, h** and **i**, are limited by lower and upper quartiles and midline indicates median; whiskers show minimum to maximum value (**i**) or extend from the box to the smallest or largest value no further than 1.5*inter-quartile range (**c, d, g** and **h**). Unpaired *t*-test with Welch’s correction, two-tailed, alpha = 0.05, significance was set as P-value < 0.05.

Our model is very well suited to explore how AD genetic risk modulates human microglial responses. As proof-of-concept, we investigated some of the major genetic risk factors for AD, hypothesizing that they would modulate microglia according to their role in AD risk, that is, *APOE2/2* should increase protective and/or decrease damaging responses, whereas *APOE4/4* and *TREM2* mutations should increase damaging or reduce protective responses^1^, to the extent that their pathological effect is restricted to microglia. We therefore tested first the effect of the clinical mutation *TREM2*^*R47H*^ in the H9 background (Table 1). We profiled 3,282 H9 (*n* = 2) and 5,845 H9-*TREM2*^*R47H*^ (*n* = 3) microglia transplanted in *App*^*NL-G-F*^ mice (Supplementary Figs. 2 and 3). The *TREM2*^*R47H*^ mutation induced a significant reduction in the fraction of cells recruited into HLA, but also an unexpected reduction of the CRM-1-cluster (Fig. 7a,b and Extended Data Fig. 9a–d). Trajectory analysis confirmed that this clinical mutant remains largely locked in a homeostatic state (Fig. 7c and Extended Data Fig. 9c,d). We compared the gene expression alterations induced by complete loss-of-function of *TREM2* and the clinical *TREM2*^*R47H*^ mutant (Fig. 7d). Both genotypes show a strong correlation (*R* = 0.66) and a common downregulation of DAM and HLA genes, which is supported by morphological evidence in *TREM2*^*R47H*^ knock-in mice showing a reduction in the number of plaque-associated microglia^38,39^. However, whereas *TREM2*^*−/−*^ retains expression of CRM genes (including *IL1B*, *CCL3L3 or CCL3L1*), *TREM2*^*R47H*^ shows only spared expression of IRM genes (such as *IFIT1*, *IFIT3*, *IFI6* or *IFITM3*). Our data indicate that *TREM2*^*R47H*^ results in an inability of microglia to engage in both HLA and CRM transcriptional programs.

Upregulation of *ApoE* in microglia is one of the main transcriptional changes induced by Aβ plaques in mouse systems^3–5^. This suggests that the *APOE* allelic variants could be important regulators of human microglia. We used a series of isogenic *APOE2/2*, *3/3* and *4/4* iPSC lines (UKBIO11-A, European Bank for induced Pluripotent Stem Cells (EBiSC)). We also obtained a second series of the *APOE* alleles in an *APOE* knockout iPSC line (BIONi010-C, EBiSC) with *APOE2/0*, *3/0* and *4/0* genotypes (Supplementary Figs. 2 and 3). Both series have very similar *APOE* expression levels (Supplementary Fig. 6) and we therefore grouped them together for our analyses. We transplanted microglial precursors from all these lines into *App*^*NL-G-F*^ mice (*n* = 3 per cell line). Unlike *TREM2*^*R47H*^ cells, clustering analysis did not reveal large differences between cells harboring the different *APOE* allelic variants. *APOE4/4*-expressing microglia show, however, a significant reduction in the proportion of cells acquiring the HLA phenotype (*P* < 0.05) and a tendency toward an increase in CRM response (*P* = 0.17; Fig. 7e,f and Extended Data Fig. 9e,f). The response of *APOE4* cells to Aβ pathology strongly correlates with that of *APOE*^−/−^ cells (Fig. 7i), suggesting that *APOE4* results in a loss-of-function phenotype in microglia. This downregulation of HLA genes is not observed in *APOE2* microglia (Fig. 7j). This is consistent with recent observations in mouse models, where microglial *APOE4* has a negative impact in the acquisition of the MGnD phenotype^40^ and interferes with complement and lysosomal pathways^41^. Trajectory analysis confirmed this and revealed that *APOE4* fails to transition toward the HLA state (Fig. 7g and Extended Data Fig. 9g,h), suggesting an inability to engage in a full response to Aβ plaques. Despite *APOE4* being the major genetic risk factor for AD, our data suggest that its cell-autonomous role in microglia is rather limited and consistent with the effect we observed in *APOE*^−/−^ microglia. As mentioned before, ApoE has a major effect on Aβ aggregation that might indirectly influence the function of microglia^34^. Nevertheless, the changes induced by *TREM2*^*R47H*^ and *APOE4* show a common impairment in activating the human-specific HLA (rather than DAM) transcriptional program and together suggest that the HLA response to Aβ plaques might be a beneficial aspect of the response of human microglia to AD pathology (Fig. 7h).

### AD risk genes are expressed in different cell states

**Fig. 8:**
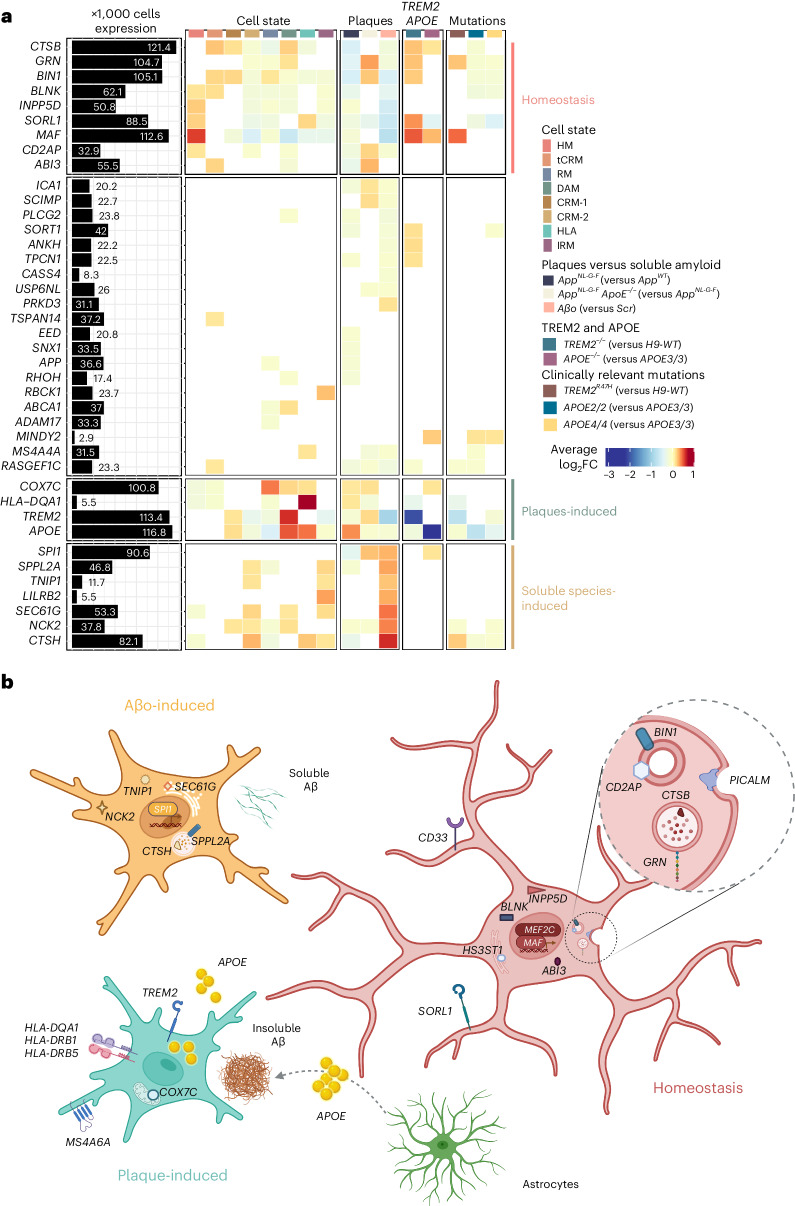
AD genetic risk genes are differentially expressed in human microglial cell states and modulated by Aβ pathology. **a**, Analysis of genome-wide association studies (GWAS) genes enrichment in xenotransplanted microglia. The black bars represent the number of cells (in thousands) with detectable expression (≥1 read per cell) for each candidate gene from Bellenguez et al.^42^. The heatmap summarizes the deregulated expression (logFC, color scale) of these genes across cell states (each cluster compared with all others), as well as after exposure to Aβ plaque pathology, upon injection of soluble Aβ aggregates or altering the genetic background of the mice or the transplanted cells. The genes are ranked in rows based on hierarchical clustering. We identify three sets of genes that display a common profile across cell states (based on their enrichment in the specific microglial phenotypic transcriptional states HM, DAM and HLA, and CRM), Aβ pathology and genetic risk, and we group these profiles as: microglial homeostasis, plaque-induced genes and soluble aggregates-induced genes. The remaining genes did not show a clear enrichment in cell states or other conditions. All differential expressions were significant after adjusting *P* values using Bonferroni correction (FDR < 0.05). Only genes that are significantly changing in at least one of the tested conditions are reported, see Supplementary Table 4 and Extended Data Fig. 10 for further details. **b**, Illustration of the complex microglial AD genetics by cell-state profiles and driven by different Aβ pathologies as found in **a** and Extended Data Fig. 10.

**Extended Data Fig. 10:**
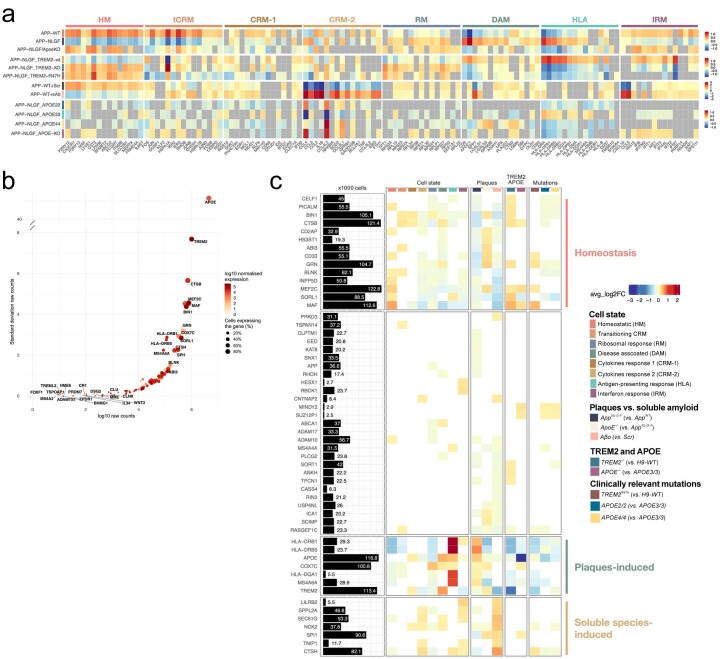
Extended analysis of GWAS genes enrichment in xenotransplanted microglia. **a**, Summary heatmap with the expression levels of the Top10 marker genes for each cluster (as in Fig. 1c), compared across all experimental groups used in this study. **b**, Expanded gene list with the canonical AD genes according to the latest GWAS study of Fig. 8a, with the addition of other genes of interest, that is the genes selected in our previous publication^14^ (see Supplementary Table 4 for the complete list). Raw counts (log scale) are reported on the x axis, while standard deviation of counts is shown on the y-axis. Dots are coloured by SCT-normalised expression (see Methods) and size reflects percentage of cells in our dataset where the gene is detected (see all values in Supplementary Table 4). All genes from (a) that are significantly changing in at least one of the tested conditions in this manuscript are reported in an extended heat map build as in Fig. 8a. The black bars represent the number of cells (in thousands) with detectable expression (>=1 read per cell) for each candidate gene. The heat map summarizes the deregulated expression (LogFC, colour scale) of these genes across cell states (each cluster compared to all others), as well as after exposure to amyloid-β plaque pathology, upon injection of soluble aggregates of Aβ, or altering the genetic background of the mice or the transplanted cells. The genes are ranked in rows based on hierarchical clustering. We identify 3 sets of genes that display a common profile across cell states (based on their enrichment in the specific microglial phenotypic transcriptional states HM, DAM and HLA, and CRM), amyloid-β pathology and genetic risk, and we group these profiles as: microglia Homeostasis, plaque-induced genes, and soluble aggregates-induced genes. The remaining genes did not show a clear enrichment in cell states or other conditions. All differential expressions were significant after adjusting *P-values* using Bonferroni correction (FDR < 0.05).

Finally, we extracted a list of 85 genome-wide association studies (GWAS) significant gene candidates (nearest gene to significant genetic loci, *P* < 5 × 10^−8^) from the most recent meta-analysis of AD complex genetics^42^. We found that almost 90% of these genes were detected in our dataset (Supplementary Table 4 and Extended Data Fig. 7a) while more than half were differentially expressed in the conditions we tested, confirming that a large part of the genetic risk for AD is harbored by microglia and that expression of these genes is modified in the context of Aβ pathology^6^ (Fig. 8a and Extended Data Fig. 10). We observed that different subsets of AD risk genes were enriched in all cell states in response to Aβ pathology. Whereas genes such as *ABI3*, *INPP5D*, *SORL1*, *GRN*, *BIN1* and *MAF* were enriched in homeostatic microglia, both the response to Aβ plaques and Aβo soluble aggregates were associated with distinctive groups of genes, including *TREM2*, *APOE*, *COX7C* and *HLA-DQA1* in DAM/HLA; and *SPl1*, *SPPL2A*, *TNIP1*, *LILRB2*, *SEC61G*, *NCK2* and *CTSH* in the response to Aβο (Fig. 8a and Extended Data Fig. 10). We also observed altered expression of subsets of these AD risk genes after introducing clinically relevant mutation *APOE4* or *TREM2*^*R47H*^. Consistent with their inability to transition toward responsive cell states, both *TREM2*^−/−^ and *TREM2*^*R47H*^ microglia showed higher expression of those risk genes present in homeostatic cells, such as *SORL1*, *GRN*, *CSTB*, *BIN1* and *MAF*; and reduced expression of the DAM and HLA genes *APOE* and *HLA-DQA1* (Fig. 8a). *APOE2/2* and *APOE4/4* microglia showed milder enrichment of risk genes when compared with *APOE3/3*, but in a similar direction and with decreased association to DAM and HLA genes (Fig. 8a and Extended Data Fig. 10). Our data show that the contribution of AD genetics to the biology of microglia is complex, likely having an impact on multiple cell states. It is interesting to see how known genetic risk factors affect the expression of other genetic risk factors in microglia, providing a strong basis for the concept of polygenic risk and providing a framework to understand its functional implications (Fig. 8b).

## Discussion

We provide a full transcriptomic characterization of the different cell states adopted by human microglia in response to Aβ pathology by using a xenotransplantation model where we engrafted stem cell-derived human microglia in the brain of the *App*^*NL-G-F*^ model of amyloid pathology^14^. Human microglia respond to AD’s multi-faceted Aβ pathology with a complex, multipronged response covering, among others, a late HLA response and a strong CRM response, which are not clearly recapitulated in mouse microglia. Even the human DAM response, which has also been observed in mouse microglia as a reaction to Aβ plaques, is only 20–25% similar to the mouse DAM response. This should be carefully considered when using preclinical models to investigate the role of blocking or boosting this putative protective cell state for potential therapeutic development. Interestingly, TREM2 and APOE have different, complementary effects in mediating human microglial activation. Similar to mouse^4,5,43^, human TREM2 appears a master inductor of DAM and HLA responses against Aβ plaques. APOE plays a more subtle role, affecting mostly the phenotypic switch from DAM to HLA.

We demonstrate here that genetic risk of AD significantly alters the response of human microglia to Aβ pathology. *APOE4* or the *TREM2*^*R47H*^ mutation hampers the HLA profile, suggesting a protective role of this response against AD. On the other hand, it is unclear how the CRM response against soluble Aβo species contributes to AD pathology. CRM is characterized by the upregulation of classical inflammatory cytokines and chemokines such as *IL1B*, *CCL2*, *CCL3*, *CCL4* and components of the *NFKB* pathway. It is also enriched with several AD risk genes. Soluble Aβ species appear early during the disease process long before Aβ plaques and have been linked to early neuronal dysfunction^44^. Our data suggest that microglia might engage with this Aβo already in the very early disease stages of AD. The question remains as to whether the upregulated cytokines and chemokines characteristic for this response affect neurons or other brain cells, inducing the cellular responses in AD that ultimately result in neurodegeneration^45^.

One strength of the current dataset appears to be that it helps to better classify the multipronged response of microglia as recorded in single nuclei isolated from AD postmortem samples. The transplantation system therefore provides a complementary tool to analyze the human-specific microglial contribution to AD. In addition, its great flexibility makes it possible to analyze the effects of genetic or pharmacological manipulation of human microglia in the context of disease in an unprecedented way. A major shortcoming of the model is the lack of adaptive immunity, which is needed to allow the xenotransplant, which at the same time may preclude the evaluation of the contribution of the adaptive immune system to the microglial responses in AD.

In conclusion, our findings unravel the complex responses of human microglia to early and late Aβ pathology in AD. The results indicate that therapeutic targeting of microglia needs to be implemented with care as it might differentially affect their cell states and modify the disease course in unpredictable ways. The xenograft model provides a good way to investigate this question and to further explore the genetics underlying the multipronged responses of human microglia to amyloid pathology in AD.

## Methods

### Mice

The *App* single knock-in mouse model (*App*^*NL-G-F*^; Takaomi Saido)^47^ does not overexpress APP as in classical APP mouse models, but contains the humanized Aβ sequence, as well as Swedish (NL), Arctic (G) and Iberian (F) mutations. *App*^*NL-G-F*^ mice accumulate Aβ plaques and suffer from learning, memory and attention impairments from 6 months onwards^47,48^. The humanized *App*^*hu/hu*^ mice (named *App*^*WT*^ in the main text) were recently generated in our laboratory to serve as controls^27^. Both strains were crossed with homozygous *Rag2*^*tm1.1Flv*^ *Csf1*^*tm1(CSF1)Flv*^ *Il2rg*^*tm1.1Flv*^ *App*^*tm3.1Tcs*^ mice (Jackson Laboratory, strain 017708) to generate the *Rag2*^−/−^ *Il2rγ*^−/−^ *hCSF1*^*KI*^ *App*^*NL-G-F*^ and the *Rag2*^−/−^ *Il2rγ*^−/−^ *hCSF1*^*KI*^ *App*^*hu/hu*^ used in this study. In total, we transplanted >150,000 cells across 11 different cell lines, and three mouse background genotypes. For all experiments, we used Total-Seq A hashing antibodies (BioLegend) so as to be able to demultiplex individual mouse replicates (Supplementary Fig. 2). Mice had access to food and water ad libitum and were housed with a 14/10-h light/dark cycle at 21 °C and 32% humidity, in groups of 2–5 animals. All experiments were conducted according to protocols approved by the local Ethical Committee of Laboratory Animals of KU Leuven (Ethische Commissie Dierproeven project no. P177/2017) following country and European Union guidelines.

### Generation of *Rag2*^*−/−*^*Il2rγ*^*−/−*^*hCSF1*^*KI*^*App*^*NL-G-F*^*ApoE*^*−/−*^ mice

*ApoE* knockout mice were generated in zygotes from homozygous *Rag2*^*tm1.1Flv*^ *Csf1*^*tm1(CSF1)Flv*^ *Il2rg*^*tm1.1Flv*^ *App*^*tm3.1Tcs*^ mice using CRISPR–Cas9 technology by targeting exon 4 of the mouse *ApoE* gene. The RNA guide 5′-CCTCGTTGCGGTACTGCCCGAGT-3′ was selected using the CRISPOR web tool. Ribonucleoproteins containing 0.3 μM purified Cas9HiFi protein (Integrated DNA Technologies), 0.3 μM CRISPR RNA (crRNA) and 0.3 μM *trans*-activating crRNA (Integrated DNA Technologies) were injected into the pronucleus of 72 embryos by microinjection in the Mouse Expertise Unit of KU Leuven. Two candidate pups were identified by PCR analysis with several primer combinations. One founder was selected for breeding and an allele with a chromosomal deletion of 335 base pairs (bp) (corresponding to 148 bp of intronic sequence and the first 187 bp of exon 4 sequence) (Extended Data Fig. 4) was selected to establish the colony. The founder mouse was backcrossed over two generations before a homozygous colony was established, which was designated *App*^*NL-G-F*^ *ApoE*^−/−^. The strain was maintained on the original C57Bl6J:BalbC background. Standard genotyping for the *ApoE* allele was performed by PCR with primers 5′-GCTCCCAAGTCACACAAGAA-3′ and 5′-CTCACGGATGGCACTCAC-3′, resulting in a 755-bp amplicon for the WT allele and a 420-bp amplicon for the *ApoE* knockout allele.

### Differentiation of microglial progenitors

UKBIO11-A, BIONi010-C and H9-WA09 and their isogenic modifications (Table 1) were differentiated into microglial precursors and transplanted following our recently published protocol, MIGRATE^26^. In brief, stem cells were plated and maintained in human Matrigel-coated six-well plates and in E8 flex media until reaching ~70–80% confluence. Once confluent, stem cell colonies were dissociated into single cells and plated into U-bottom 96-well plates at a density of ~10,000 per well in mTeSR1 medium with BMP4 (50 ng ml^−1^), VEGF (50 ng ml^−1^) and SCF (20 ng ml^−1^) for 4 d. On day 4, embryoid bodies were transferred into six-well plates (~20 embryoid bodies per well) in X-VIVO (+supplements) medium supplemented with SCF (50 ng ml^−1^), M-CSF (50 ng ml^−1^), IL-3 (50 ng ml^−1^), FLT3 (50 ng ml^−1^) and TPO (5 ng ml^−1^) for 7 d with a full change of medium on day 8. On day 11, differentiation medium was replaced with X-VIVO (+supplements) with FLT3 (50 ng ml^−1^), M-CSF (50 ng ml^−1^) and GM-CSF (25 ng ml^−1^). On day 18, human microglial precursors were collected and engrafted into P4 mouse brains (0.5 million cells per pup) as previously described. Before transplantation, mouse microglia were depleted by inhibiting CSF1 receptor (CSF1R) with BLZ945 (dose of 200 mg kg^−1^) at P2 and P3 as previously described^14,26^. To confirm the biological activity of known essential mouse cytokines on human microglia, we cultured microglial progenitors in TIC medium^49^ for 9 d, supplemented with either hCSF1, hIL-34, hTGFb and hCX3XR1, or hCFS1, mIL-34, mTGFb and mCX3CR1, and measured the expression of several microglial makers by quantitative PCR (qPCR). We did not find any differences in the levels of P2RY12, CX3CR1, C1Q, HEXB, TGFbR1 and TREM2 (Supplementary Fig. 1a).

### Genetic modification of stem cell lines

Generation of *TREM2*^*−/−*^ and *TREM2*^*+/R47H*^ from H9-WT (WA09) human embryonic stem cells was done as described by Claes et al.^46^. Briefly, the *TREM2*^*+/R47H*^ CRISPR–Cas9 nickases and the two guide RNAs (gRNAs) (gRNA A and B) that target exon 2 of *TREM2* nearby the location of *R47H* (*G*>*A*) and a genomic *TTAA* were purchased from Addgene. A donor plasmid was made, comprising homology arm 1 (HA1) of *TREM2* (with the *R47H* mutation), a selection cassette (CAGG promoter, HYG/TK, green fluorescent protein) and HA2 of *TREM2* exon 2. To create *TREM2*^−/−^ human pluripotent stem cells (hPSCs), a CRISPR–Cas9, gRNA B and the same donor plasmid were used. To create *TREM2*^*+/R47H*^ hPSCs, 2 × 10^6^ single cells of the heterozygously targeted clone were nucleofected with 4 μg of piggyBac transposase plasmid and negative selection with fialuridine, also known as 1-(2-deoxy-2-fluoro-1-d-arabinofuranosyl)-5-iodouracil (FIAU) (1:8,000–1:2,500; 0.5 mM in water), was applied to select for cells wherein the selection cassette was removed. Of note, the H9-WT line from which the *TREM2*^*+/R47H*^ and *TREM2*^*−/−*^ lines were created carries an APOE ε3/ε4 genotype.

To get the gRNA into the cells, nucleofection was performed. Briefly, 2 × 10^6^ single-cell H9s were preincubated with Revitacell (Life Technologies) and nucleofected using the Amaxa Nucleofector II on setting F16 with 2.5 μg of CRISPR–Cas9, 2.5 μg of gRNA (A and) B and 5 μg of donor template to create TREM2^−/−^ hPSCs. Selection was initiated after 2–3 d with 25–150 μg ml^−1^ Hygromycin B (Sigma-Aldrich) and maintained for 10–15 d. Recombinant colonies were manually picked and expanded for further characterization.

**Table:** 

gRNA A	F: CACCGACCCAGGGTATCGTCTGTGA	R: AAACTCACAGACGATACCCTGGGTC
gRNA B	F: CACCGCACTCTCACCATTACGCTG	R: AAACCAGCGTAATGGTGAGAGTGC

The H9 (WA09) embryonic stem cell line was also modified to express the CAS9 in a doxycycline-dependent manner (iCas9), using TALENS at the AAVS1 locus to facilitate the generation of targeted gene deletion. H9-iCas9 was used only in libraries 14 and 16 and doxycycline was never administered.

### *APOE* expression using qPCR

The levels of *APOE* expression in the grafted stem cell lines were checked by collecting cell pellets. Using the RNeasy Micro Kit, RNA was extracted according to manufacturer’s instructions and the RNA was reverse transcribed with the High-Capacity cDNA Reverse Transcription Kit. A qPCR was performed with SensiFast SYBR reagent and custom-made primers for *GAPDH* (FW: tcaagaaggtggtgaagcagg; RV: accaggaaatgagcttgacaaa) and *APOE* (we average the level of expression of multiple primers spanning the whole gene, see table below).

**Table:** 

hAPOE_1_F	F: TAGAAAGAGCTGGGACCCT	R: CACAGAACCTTCATCTTCCT
hAPOE_2_F	F: GTTGCTGGTCACATTCCTG	R: GCAGGTAATCCCAAAAGCGA
hAPOE_3_F	F: CTGGGTCGCTTTTGGGATTA	R: GTCAGTTGTTCCTCCAGTTC
hAPOE_4_F	F: AATCACTGAACGCCGAAG	R: TTATTAAACTAGGGTCCACC
hAPOE_qPCR_F	F: GTTGCTGGTCACATTCCTGG	R: GCAGGTAATCCCAAAAGCGAC

### Soluble Aβ preparation and intracerebroventricular injections

Soluble Aβ aggregates (10 μM) or scrambled peptides (10 μM) were prepared as previously^14,50^. Briefly, recombinant Aβ (1–42) or scrambled peptides were thawed during 30 min at room temperature and dissolved in hexafluoroisopropanol at 2 mg ml^−1^. Hexafluoroisopropanol was fully evaporated with a gentle stream of N_2_ gas and resulting peptides were dissolved in dimethylsulfoxide at 2 mg ml^−1^. Dimethylsulfoxide medium was removed using HiTrap Desalting column 5kD and peptides were eluted in Tris-EDTA buffer. Of note, Tris-EDTA buffer was composed of 50 mM Tris buffer and 1 mM EDTA at pH 7.5. Tris-EDTA-eluted Aβ or scrambled peptides were quantified using Bradford assay before aggregation. Peptides were left to aggregate for 2 h at room temperature. After 2 h, Aβ (1–42) or scrambled aggregates were diluted to a final concentration of 10 μM in Tris-EDTA buffer, snap frozen and stored at −80 °C. Following a similar approach as previously described^14^, at 12 weeks of age, *App*^*WT*^ mice engrafted with the full isogenic series of UKBIO11-A or BIONi010-C were anesthetized with isoflurane and injected intracerebroventricularly with 5 μl of soluble aggregates of Aβ (10 μM) or scrambled peptides (10 μM). Stereotactic coordinates from Bregma: anteroposterior: −0.22 mm; mediolateral: −1 mm; dorsoventral: −2.74 mm. After surgery, mice were placed on a thermal pad until recovery. At 6 h after injection, *App*^*WT*^ mice were euthanized, and human microglia were isolated using FACS for transcriptomics analysis.

### Human microglia isolation from mouse brain for single-cell transcriptomics

At 6–7 months of age *App*^*NL-G-F*^, *App*^*NL-G-F*^ *ApoE*^−/−^ and *App*^*WT*^ mice xenotransplanted with H9, UKBIO11-A, BIONi010-C (Table 1) and their isogenic modifications were killed with an overdose of sodium pentobarbital and immediately perfused with ice-cold 1 × DPBS (Gibco, Cat. no. 14190-144) supplemented with 5 U of heparin (LEO). After perfusion, one hemisphere of each mouse brain without cerebellum and olfactory bulbs was placed in FACS buffer (1 × DPBS, 2% FCS and 2 mM EDTA) + 5 μM actinomycin D (ActD; Sigma, Cat. no. A1410-5MG) for transcriptomics. Brains were mechanically and enzymatically dissociated using Miltenyi Neural Tissue Dissociation Kit P (Miltenyi, Cat. no. 130-092-628) supplemented with 5 μM ActD. Next, samples were passed through a 70-μm strainer (BD2 Falcon), washed in 10 ml of ice-cold FACS buffer + 5 μM ActD and spun at 300*g* for 15 min at 4 °C. Note that 5 μM ActD was kept during collection and enzymatic dissociation of the tissue to prevent artificial activation of human microglia during the procedure as previously reported^12^. ActD was removed from the myelin removal step to prevent toxicity derived from long-term exposure. Following dissociation, myelin was removed by resuspending pelleted cells in 30% isotonic Percoll (GE Healthcare, Cat. no. 17-5445-02) and centrifuging at 300*g* for 15 min at 4 °C. Accumulating layers of myelin and cellular debris were discarded and Fc receptors were blocked in FcR blocking solution (1:10, Miltenyi, Cat. no. 130-092-575) in cold FACS buffer for 10 min at 4 °C. Next, cells were washed in 5 ml of FACS buffer and pelleted cells were incubated with the following antibodies: PE-Pan-CD11b (1:50, Miltenyi, Cat. no. 130-113-806), BV421-mCD45 (1:500, BD Biosciences, Cat. no. 563890), APC-hCD45 (1:50, BD Biosciences, Cat. no. 555485), Total-Seq A cell hashing antibodies (1:500, BioLegend) and viability dye (1:2,000, eFluor 780, Thermo Fisher Scientific, Cat. no. 65-0865-14), in cold FACS buffer during 30 min at 4 °C. After incubation, cells were washed, and the pellet was resuspended in 500 μl of FACS buffer and passed through a 35-μm strainer before sorting. For sorting, the cell suspension was loaded into the input chamber of a MACSQuant Tyto Cartridge and human cells were sorted based on CD11b and hCD45 expression at 4 °C (Supplementary Fig. 1). FACS data were analyzed using FCS Express 7 software.

### Histology

When killing and collecting brains of mice for single-cell sequencing, one hemisphere of *App*^*NL-G-F*^, *App*^*NL-G-F*^ *ApoE*^−/−^ *and App*^*WT*^ mice xenotransplanted with H9, UKBIO11-A and BIONi010-C was also preserved and postfixed in 4% PFA overnight at 4 °C. After 24 h, PFA was removed and they were washed and kept in 1 × DPBS at 4 °C until further processing. For sectioning, olfactory bulbs and cerebellum were discarded and brains were cut coronally (40-µM thickness) with a vibrating microtome (Leica). Each sample was collected under free-floating conditions in a series of six sections and stored in cryoprotectant solution (40% PBS, 30% ethylene glycol, 30% glycerol) at −20 °C. For staining, sections are washed in 1 × DPBS and permeabilized for 15 min at room temperature in PBS with 0.2% Triton. After permeabilization, sections were stained with X-34 staining solution (10 µM X-34 (Sigma-Aldrich), 20 mM NaOH (Sigma-Aldrich) and 40% ethanol) for 20 min at room temperature. Sections were washed several times with 40% ethanol for 2 min and with PBS + 0.2% Triton for 5 min. For the staining of microglia with anti-hP2RY12 (HPA014518, Sigma-Aldrich, 1:2,000), CD9 (312102, BioLegend, 1:100), FTH1 (PA5-1905, Invitrogen, 1:500) and HLA antibodies (ab7856, Abcam, 1:200), sections were blocked with 5% normal donkey serum in PBS + 0.2% Triton for 1 h at room temperature. For the costaining of CD9/FTH1 with P2RY12, primary antibody incubation was done overnight at 4 °C. For the HLA staining, signal was enhanced using a Tyramide SuperBoost kit (B40915, Thermo fisher) according to the manufacturer’s instructions. Briefly, after overnight incubation with HLA primary antibody, sections were incubated with a poly-HRP-conjugated secondary antibody. Tyramid solution was then added to the slices for 5 min and they were washed in PBS + 0.2% Triton after the reaction was stopped. The HLA sections were later costained with P2RY12 as previously described. Secondary antibodies were incubated for 1 h at room temperature. Finally, sections were mounted with Mowiol mounting medium (Sigma-Aldrich) or DAKO mounting medium (Agilent). Images at ×4 and ×20 magnification were taken on a Nikon A1R Eclipse confocal microscope. To measure the shift in microglial cell states at the site of Aβ plaques, we used a modified Sholl analysis where the fluorescent intensity of microglial markers P2RY12, CD9 and HLA was measured through concentric rings (annuli) of increasing diameter surrounding the X-34 plaque center. The analysis was performed using ImageJ software after determining a threshold for background correction. Intensities of each channel were scaled for comparison using *z*-score normalization. Intensity over distance (µm) was plotted using Loess nonparametric regression in R with estimated standard error for each predicted value. For comparison of intensities near and distant from the plaque center, the means of the inner and outer three annuli were independently calculated. Bar plots were generated in Prism v.10.

### Single-cell library preparation and sequencing

For single-cell RNA sequencing, 15,000–20,000 human microglia (CD11b^+^, hCD45^+^) from each mouse were sorted on the MACSQuant Tyto (Supplementary Fig. 1) and diluted to a final concentration of 1,000 cells per µl. Since all the samples were individually hashed using Total-Seq A cell hashing antibodies, 2,000 human microglia per animal were pooled and loaded onto the Chromium Next GEM Chip G (PN no. 2000177). The DNA library preparations were generated following the manufacturer’s instructions (CG000204 Chromium Next GEM Single Cell 3′ Reagent Kits v3.1). In parallel, the hashtag oligo libraries were prepared according to the manufacturer’s instructions (BioLegend, Total-Seq A Antibodies and Cell Hashing with 10x Single Cell 3′ Reagent Kit v3 3.1 Protocol) using 16 cycles for the index PCR. A total of 20 libraries containing 95 biological replicates were sequenced, targeting a 90% messenger RNA and 10% hashtag oligo library (50,000 reads per cell), on a HiSeq4000 or NovaSeq6000 (Illumina) platform with the recommended read lengths by the 10X Genomics workflow.

### Statistics and reproducibility

Statistical analysis of the distribution of different experimental groups across clusters was performed using each mouse as a single replicate. Normality and equal variance were tested, and the data were normalized if needed. We used both *t*-test and one-way analysis of variance (ANOVA) when comparing two or more than two groups, respectively. Statistical significance was set at *P* < 0.05, and multiple comparisons Bonferroni was applied when necessary. No statistical methods were used to predetermine sample sizes, but our sample sizes are similar to those reported in previous publications^3,14^. All the mice were randomized across the different experimental groups. Data collection and analysis were performed by blind researchers. Immunohistochemical data were repeated at least five times with similar results.

### Analysis of single-cell RNA sequencing datasets

#### Alignment and software

The raw BCL files were demultiplexed and aligned by Cellranger (v.3.1.0) against the human genome database (hg19, Ensembl 87). Raw count matrices were imported in R (v.4.1.3) for data analysis. Datasets were analyzed using the Seurat R package pipeline (v.4.0.1). For specific statistical tests and visualizations, we also used GraphPad Prism v.9.0, Python, R and Bioconductor.

#### Quality control of cells and samples

For each library included in this study, we excluded low-quality cells (poorly sequenced, damaged or dead cells) by filtering out cells with <1,000 reads or <100 genes detected. We also excluded cells with >15% of reads aligning to mitochondrial genes. Doublets were first excluded by removing cells with a number of reads or genes more than 3 s.d. from the library mean (Supplementary Fig. 2a). Doublets removal was further refined with cell hashing information by using Seurat’s function MULTIseqDemux() to assign cells to singlets, doublets or negatives (Supplementary Fig. 2b). Only singlets were retained, as negative cells cannot be demultiplexed and assigned with certainty to the sample of origin. For one library out of 20 sequenced for this study (library 9), the counts related to one hash (sample MG452) were high across all samples in the library and MULTIseqDemux() failed to demultiplex many cells. We used the function HTODemux() instead to demultiplex library 9 which performed better. Sample MG452 was entirely removed in further quality control steps (see below). Genes detected in fewer than three cells were excluded from the count matrices. At this step, when quality control of single cells was completed, the dataset consisted of 154,624 cells across 101 independent mice and 20 sequencing libraries (Supplementary Fig. 2d,c and Tables 5–7). For detailed sequencing statistics per library see Supplementary Tables 1–8.

#### Normalization and integration

After quality control, each library was individually normalized and scaled using SCTransform(). For all libraries, we selected the 3,000 most variable features for downstream integration. We determined a list of common integration anchors across libraries with FindIntegrationAnchors() that we used as an input for integration. To integrate all the libraries, we used the IntegrateData() function of the Seurat package to correct for any potential library batch effect. Integrated matrix was used for downstream analysis (Supplementary Fig. 2c,d).

In the integrated dataset, we performed principal components analysis and found that the highest variability in the dataset was explained by the separation of CAMs from the microglial cell states, while integrated sequencing libraries did not show any batch effects in the dataset (Supplementary Fig. 2c,d). We selected 27 dimensions for dimensionality reduction by Uniform Manifold Approximation and Projection (UMAP), which we performed with the RunUMAP() function. To produce the initial UMAP as in Supplementary Fig. 3a we used the following parameters in RunUMAP(): dims = 1:27, n.neighbors = 30L, n.epochs = 200, min.dist = 0.01. To identify clusters, we first used the function FindNeighbors() (parameters for Supplementary Fig. 3a: dims = 1:20, k.param = 100, nn.method = “annoy”, annoy.metric = “cosine”) and then performed unbiased clustering by using FindClusters() (parameters for Supplementary Fig. 3a: resolution = 0.6, n.iter = 1000, n.start = 10, algorithm = 1, group.singletons = T). This led to the identification of 14 clusters, ten of which represented unique microglial/myeloid cell-state identities, three of which were merged into one homeostatic cluster for their overlapping transcriptomic signature and one of which resulted in a small low-quality cell cluster. The specific parameters used for UMAP and clustering were defined after assessment of a wide range of possible parameters, which were evaluated in light of cell-state annotations and differential expression results. We start from underclustering and we progressively increase the resolution by identifying further heterogeneity in the data, but we prevent overclustering by assessing that high resolutions lead to the definition of extra clusters that do not significantly differ in gene expression from the existing ones. Cell states were annotated by means of differential expression (FindAllMarkers() function for overall differential expression, FindMarkers() for side by side comparison) and using the AddModuleScore() function with a large number of published datasets, gene ontology categories, pathways and signatures as input^3,4,7,9,14,20^ (Supplementary Fig. 3b). Out of 14 clusters, nine clustered together and showed high expression of human microglial genes (*P2RY12*, *CX3CR1*, P2RY13 and so on). Three of these clusters were merged into an HM cell state for their common signature, while unique cell states were assigned to the other microglial clusters (Supplementary Fig. 3b,c, see main text and Fig. 1b–d for details). The remaining five clusters were enriched in nonmicroglial markers. Out of those, one small population clustered away from the main microglial clusters and expressed high levels of CAM markers (9,645 cells, marked by *CD163*, *MRC1*, *RNASE1*; Supplementary Fig. 3c,d) as previously described by Mancuso et al.^14^. In total, 3,396 cells clustered apart and expressed proliferation markers (high in *TOP2A*, *MKI67, STMN1*; Supplementary Fig. 3c,d). Although the dataset was previously quality controlled, a population of Low Quality/Doublets (3,517 cells) was still present. A small cluster of secretory cells was defined by sharing the ‘secretory’ signature previously identified also in Hasselman et al.^7^ (1,156 cells, high in *AGR2*, *MNDA*; Supplementary Fig. 3c,d). Finally, we defined a small cluster of other myeloid cells characterized by low expression of microglial homeostatic genes and expression of macrophage/monocyte markers (*CD14*, *NEAT1*, *MAFB*) and pro-inflammatory markers (*IL1B*, *CCL2*, *CCL3*) (3,078 cells; Supplementary Fig. 3c). The top differentially expressed genes from each defined cell state confirmed the unique signatures and no overclustering (top 20 marker genes per cluster are visualized in Supplementary Fig. 3c).

#### Microglia subsetting, re-clustering and annotation

For the final analysis of high-quality isolated human microglia, we subsetted out other myeloid and low-quality clusters (CAM, Other Myeloid, Secretory, Proliferating and Doublets/Low Quality). After trimming, 127,755 human microglia were retained for downstream analysis. Using the previously integrated dataset, we performed principal components analysis and selected 30 dimensions for dimensionality reduction by UMAP, as described above. No library-dependent batch effect was observed (Supplementary Fig. 2d). To produce the final UMAP as in Fig. 1b, we used the following parameters in RunUMAP(): dims = 1:30, n.neighbors = 30L, n.epochs = 500, min.dist = 0.05. To identify clusters, we first used the function FindNeighbors() (parameters for Fig. 1b: dims = 1:20, k.param = 100, nn.method = “annoy”, annoy.metric = “cosine”) and then performed unbiased clustering by using FindClusters() (parameters for Fig. 1b: resolution = 0.7, n.iter = 1000, n.start = 10, algorithm = 1, group.singletons = T). This led to the identification of 11 clusters, four of which were merged into an HM cell state for their overlapping transcriptomic signature (Fig. 1b,c and Supplementary Fig. 3c), and the other seven represented cell states defined by unique or transitory profiles. With the higher resolution provided by the microglia subclustering, we could identify two distinct CRM populations (CRM-1 and CRM-2) that were initially grouped together (Supplementary Fig. 3a,c) but actually represent two consecutive stages on the same phenotypic trajectory, and which can be differentially modified by Aβ pathologies and genetic backgrounds (Figs. 1b–d, 3a,b, 5d–f and 7). The specific parameters used for UMAP and clustering were defined after assessment of a wide range of possible parameters, which were evaluated in light of cell-state annotations, differential expression results and sample distributions. Cell-state annotation was performed as described for the full dataset, by means of iterative clustering, differential expression and signature scores (Fig. 1c and Supplementary Fig. 4b). We finally defined eight microglial cell states that included HM, CRM-1 and CRM-2, IRM, DAM, HLA, RM and tCRM (Fig. 1b–d). The expression profiles of the top differentially expressed genes from each defined cell state (top ten markers on heatmap in Fig. 1d, top three markers on UMAP in Supplementary Fig. 4c and all markers of differential expression statistics in Supplementary Table 8) and the signature scores calculated with AddModuleScore() (Supplementary Fig. 4a) confirmed the unique transcriptomic profiles of these clusters and that no overclustering was performed. We excluded from this dataset six mice that showed signs of infection, extremely low cell numbers and/or mice with the vast majority of cells mapping to one unique cell state. The final high-quality microglia dataset consisted of 127,755 cells from 95 independent mice and 20 sequencing libraries (Supplementary Fig. 2d,e). To extend our pseudotime analysis (Fig. 3 and Extended Data Figs. 4 and 5), we added three sequencing libraries with 10,822 single microglial cells across 11 independent mice. The total final dataset therefore includes 138,577 single cells from 106 mice and 23 sequencing libraries. For detailed sequencing statistics per library see Supplementary Table 5. For the number of sequenced cells per replicate/condition and all metadata of this study see Supplementary Tables 6 and 7.

#### Differential expression

Differentially expressed genes were found by applying the FindAllMarkers() function for overall differential expression between each cluster and the rest of the dataset and FindMarkers() for side by side comparisons of two groups, both from the Seurat R package. All the reported comparisons in the manuscript were performed with the following parameters: assay = “SCT”, test.use = “wilcox”, min.pct = 0.01, logfc.threshold = 0.1. We used the Wilcoxon rank-sum test to calculate *P* values. We performed differential expression on the ‘SCT’ assay calculated from SCTransform(), since Pearson residuals resulting from regularized negative binomial regression effectively mitigate depth-dependent differences in differential expression, as described by Hafemeister and Satija^51^. We tested only genes that were detected in a minimum fraction of 1% in either of the two populations. We limited testing to genes that showed, on average, at least 0.1-fold difference (log-scale) between the two groups of cells. Only genes with their adjusted *P* < 0.05 (post hoc, Bonferroni correction) were considered as significant. The complete list of differentially expressed genes from all comparisons is displayed in Supplementary Tables 1, 3 and 8.

#### Pseudotime

Pseudotime analysis was performed in the final human microglia dataset to infer the phenotypic transitions happening between the different microglial cell states. Unsupervised single-cell trajectory analysis was performed with Monocle 3, an algorithm that allows us to learn the sequence of gene expression changes each cell must go through as part of a dynamic biological process. We used SeuratWrappers to convert our microglial Seurat object into a Monocle object with as.cell_data_set(). We kept the UMAP embeddings previously calculated with RunUMAP() to estimate the phenotypic transitions between our annotated cell states. We ran cluster_cells() and learn_graph() (parameters used: close_loop = T, learn_graph_control = list(36an.k = 100, prune_graph = TRUE, orthogonal_proj_tip = F, minimal_branch_len = 50)) to learn the trajectory. To infer how resting microglia transition into reactive cell states, we set the roots of the trajectory with order_cells() by selecting the ten most homeostatic cells in our dataset (based on our previously defined HM signature score), to avoid limiting the selection of the root to a manually picked single cell and to account for heterogeneity of the HM cluster. Similar trials that set the origin of the trajectory to different HM cells led to comparable results, always identifying the main axes of phenotypic transitions described in Fig. 3a. To generate the trajectory displayed in Extended Data Figs. 4 and 5 including the 3-month data, we used the following parameters for learn_graph(): close_loop = F, learn_graph_control = list(rann.k = 100, prune_graph = TRUE, proj_tip = F, branch_len = 20), and we set the roots of the trajectory with order_cells() by selecting the five most homeostatic cells in the dataset.

#### Gene name conversion from human to mouse orthologs

Mouse to human orthologs tables were downloaded from Ensembl/Biomart (release 106). For the analysis reported in Extended Data Fig. 3c,d and Supplementary Table 2, mouse genes were converted to human genes with the most conservative criteria: if multiple orthologues of a mouse gene existed in human, the one with highest log_2_FC (fold change) in human DAM was selected. If multiple mouse genes converted to the same human gene, the one with highest log_2_FC in mouse DAM was selected. The overlap shown is therefore the highest that it is possible to achieve and can only be an overestimation. Note that even with the most inclusive criteria, the overlap between human and mouse DAM was very limited, and any other stricter approach (such as considering only bidirectional one-to-one orthologs) would find only lower correlations while losing information from the mouse genes.

### Data exclusion

We excluded six mice from this dataset that showed signs of infection or extremely low cell numbers, and/or mice with most cells mapping to one unique cell state.

### Reporting summary

Further information on research design is available in the Nature Portfolio Reporting Summary linked to this article.

## Online content

Any methods, additional references, Nature Portfolio reporting summaries, source data, extended data, supplementary information, acknowledgements, peer review information; details of author contributions and competing interests; and statements of data and code availability are available at 10.1038/s41593-024-01600-y.

### Supplementary information

Supplementary Information Supplementary Figs. 1–7.

Reporting Summary

Supplementary Table 1 Differential expression between microglial cell states. Differential expression results comparing each cell state in Fig. 1b with all the others identify specific markers of microglial cell states reacting to amyloid pathology. We used the Wilcoxon rank-sum test to calculate *P* values. We tested only genes that are detected in a minimum fraction of 1% in either of the two populations. We limited testing to genes that show, on average, at least 0.1-fold difference (log-scale) between the two groups of cells. Only genes with their adjusted *P* value < 0.05 (post hoc, Bonferroni correction) were considered as significant.

Supplementary Table 2 Overlap of human and mouse DAM markers. List of DAM marker genes defined in this study and in other transplantation, human postmortem and mouse studies, as compared in Extended Data Fig. 3d.

Supplementary Table 3 Differential expression between all tested conditions. Differential expression results comparing side by side all the conditions tested in this study, including genotype of the host, type of injection and *TREM2*/*APOE* mutations. We used the Wilcoxon rank-sum test to calculate *P* values. We tested only genes that are detected in a minimum fraction of 1% in either of the two populations. We limited testing to genes that show, on average, at least 0.1-fold difference (log-scale) between the two groups of cells. Only genes with their adjusted *P* value < 0.05 (post hoc, Bonferroni correction) were considered as significant.

Supplementary Table 4 Alzheimer’s disease GWAS gene expression in human microglial cell states. The table reports how the list of GWAS genes was compiled from the most recent studies, the expression levels of the risk genes in our dataset as in Extended Data Fig. 10b and their differential expression across cell states and tested conditions as in Fig. 8a and Extended Data Fig. 10c.

Supplementary Table 5 Statistics and QC of the sequencing libraries. The table reports summary statistics such as average number of genes, reads and percentage of reads mapping to mitochondrial genes for each of the 23 sequencing libraries included in this study. The percentage of cells removed by applying our QC criteria is also indicated.

Supplementary Table 6 Metadata by transplanted mouse replicate. Detailed metadata of the 106 transplanted mice used in this study, including genotype of the host, genotype of the transplanted human cells, age, sex and type of injection. The number of sequenced cells for each tested condition is also indicated.

Supplementary Table 7 Metadata by single cells. Detailed metadata of the single cells sequenced in this study, including all metadata as in Supplementary Table 2, the assigned cell state and the UMAP coordinates as in Fig. 1b.

Supplementary Table 8 Differential expression between cell states before removal of nonmicroglial cells. Differential expression results comparing each cell state in Supplementary Fig. 3a with all the others identify markers of nonmicroglial cells excluded from this study. We used the Wilcoxon rank-sum test to calculate *P* values. We tested only genes that are detected in a minimum fraction of 1% in either of the two populations. We limited testing to genes that show, on average, at least 0.1-fold difference (log-scale) between the two groups of cells. Only genes with their adjusted *P* value < 0.05 (post hoc, Bonferroni correction) were considered as significant.

## Data Availability

Data generated in this study are available at the Gene Expression Omnibus (GEO) database with accession number GSE216999 https://www.ncbi.nlm.nih.gov/geo/query/acc.cgi?acc=GSE216999 (ref. ^52^). An online platform for user-friendly access and visualization of the data shown in this study is available as a beta version both in the De Strooper lab website (data.bdslab.org/Mancuso2022) and in the Mancuso lab website (mancusolab.bioinf.be/ShinyRenzoAlfaBeta/). Other datasets included in the manuscript can be found at GEO (Mancuso et al., GSE137444; Sala Frigerio et al., GSE127893; Hasselman et al., GSE133433; Gerrits et al., GSE148822; Sayed et al., GSE183068; Zhou et al., GSE140511; Keren-Shaul et al., GSE98969; Friedman et al., GSE89482). The raw data files of Olah et al. are available through Synapse (https://www.synapse.org/#!Synapse:syn21438358). The list of GWAS risk genes for Fig. 8a was obtained according to the criteria of nearest protein coding genes to SNPs as identified in ref. ^42^ (Supplementary Table 4). The list of GWAS risk genes for Extended Data Fig. 10b,c was obtained as the union of candidate genes from Bellenguez et al. and a candidate gene list selected in our previous publication^14^ (see Supplementary Table 4 for complete list).

## Code availability

The code used for the transcriptomic and imaging data analysis is available upon request.
