# Supplementary material for: Xenografted human microglia display diverse transcriptomic states in response to Alzheimer’s disease-related amyloid-β pathology
Source: Nat Neurosci. 2024 Mar 27;27(5):886–900. doi: 10.1038/s41593-024-01600-y (PMC11089003; doi:10.1038/s41593-024-01600-y)
Supplement: Supplementary file 1 — Supplementary Figs. 1–7. [file 41593_2024_1600_MOESM1_ESM.pdf]

nature neuroscience

Article https://doi.org/10.1038/s41593-024-01600-y

Xenografted human microglia display diverse transcriptomic states in response to Alzheimer's disease-related amyloid-β pathology

In the format provided by the authors and unedited

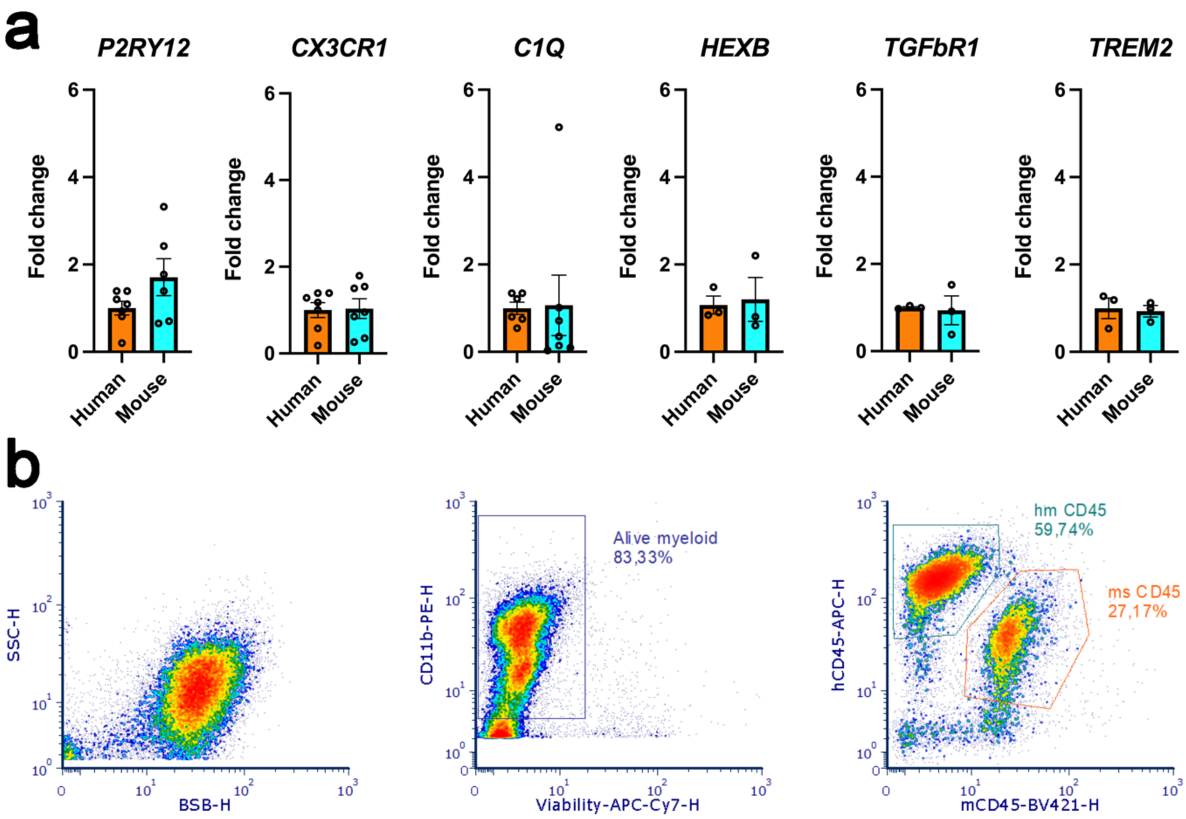

Supplementary Fig. 1. (a) Reactivity of mouse cytokines in human microglia. Comparison of mRNA expression levels in human microglia cultured with either hCSF1+hIL34+hTGFβ+hCX3CR1 (Human) or hCFS1+mIL34+mTGFb+mCX3CR1 (Mouse), measured by qPCR. Human CSF1 is used across both conditions as we transplant the cells in $hCSF1^{KI}$ mice (bar plots represent the mean +/- SEM; unpaired t-test, two-tailed, alpha = 0.05, significance set as P-value <0.05). **(b) Gating strategy for the isolation of xenotransplanted human microglia from the mouse brain.** Human cells were sorted according to the expression of CD11b+ hCD45+ whereas mouse cells were defined as CD11b+ hCD45- mCD45+. Note that we applied a trigger (=threshold) on CD11b+ events, therefore all CD11b negative cells are not displayed.

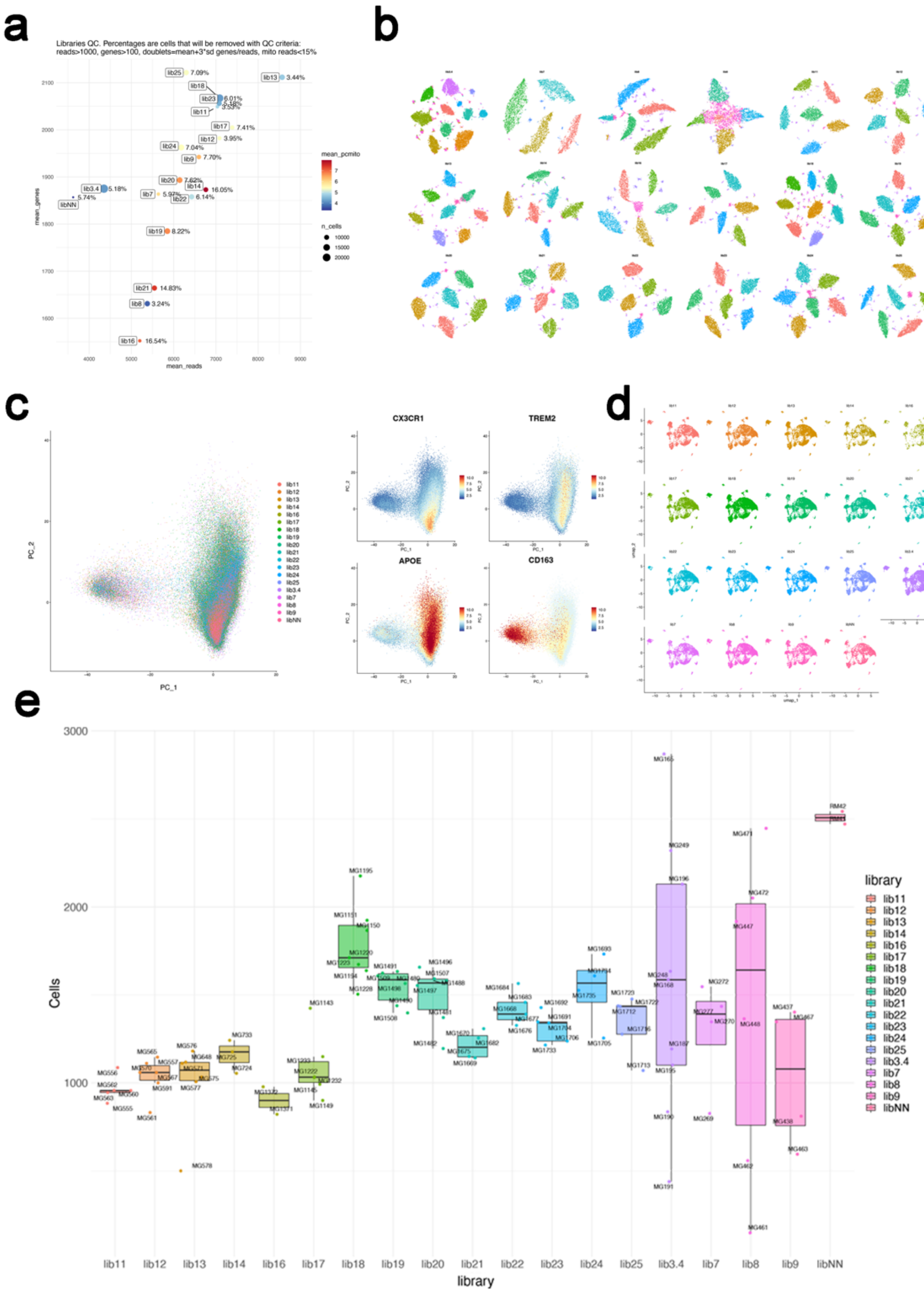

Supplementary Fig. 2. Overview of all the cDNA libraries used in single cell RNA-seq experiments. a, Basic quality control of the libraries including number of sequenced cells (size of the dot), mean number of genes (x-axis) and reads (y-axis), as well as mean percentage of reads mapping to mitochondrial genes (colour of the dot). Percentage labels indicate the proportion of cells per library that did not pass QC based on reported criteria (see also Methods). **b,** Cell-hashing counts-based tSNE plots showing the total-seq A barcoded antibody demultiplexing. Each tSNE represents a library, whereas each cluster is an individual mouse assigned to a cell hash. Small clusters are unassigned cells or remaining doublets from previous QC. **c,** First and second principal component (PC) used for the clustering analysis shown in Supplementary Fig. 3a. Libraries completely overlap after data integration, and most of the dataset variability (PC1) is explained by the separation of CAMs (*CD163*) from microglia clusters (*CX3CR1, TREM2, APOE*) (see Supplementary Fig. 3a and Methods). **d,** Individual UMAP plots as in Supplementary Fig. 3a showing the distribution of cells from each single library. **e,** Final number of cells per mouse and per library (box plots are limited by lower and upper quartiles and midline indicates median; whiskers extend from the box to the smallest or largest value no further than 1.5*inter-quartile range. Each box represents a library, whereas each dot represents an individual mouse replicate labelled with MG unique IDs. Lib NN corresponds to the data set in Mancuso et al. 2019[14]).

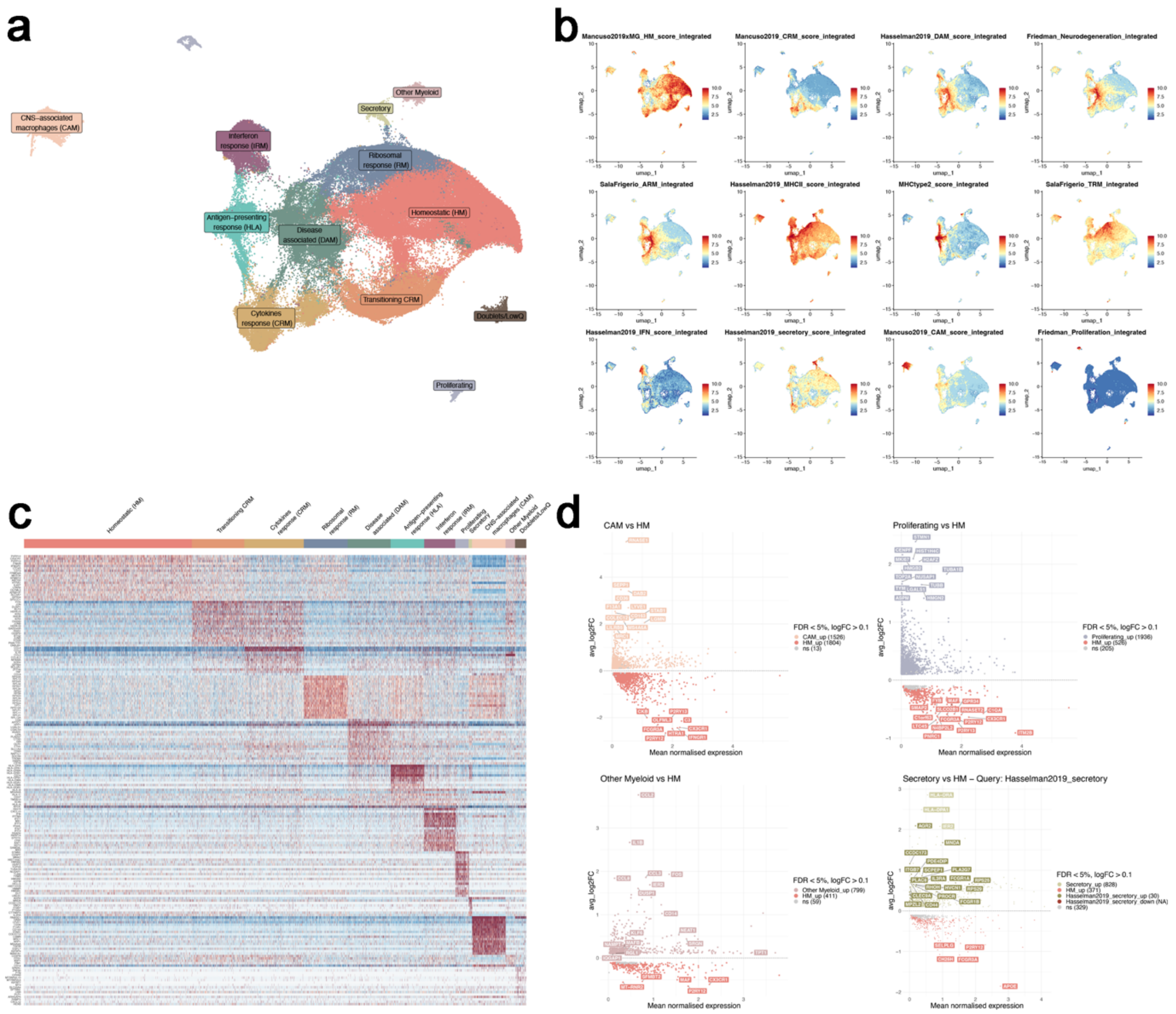

Supplementary Fig. 3. Extended clustering, preparation of the datasets for analysis and cell type/state annotations. a, UMAP plot of the 154,624 cells passing quality control, coloured by annotated cell states before removal of CAMs, other myeloid, low quality and proliferating clusters. **b,** UMAP plots as in **a**, coloured by the combined level of expression of groups of genes that characterise distinct microglial and peripheral cell states. **c,** Heatmap displaying the top20 most upregulated genes in each cluster as in **a**. **d,** MA plots with direct comparisons between clusters excluded from the analysis and homeostatic microglia (HM).

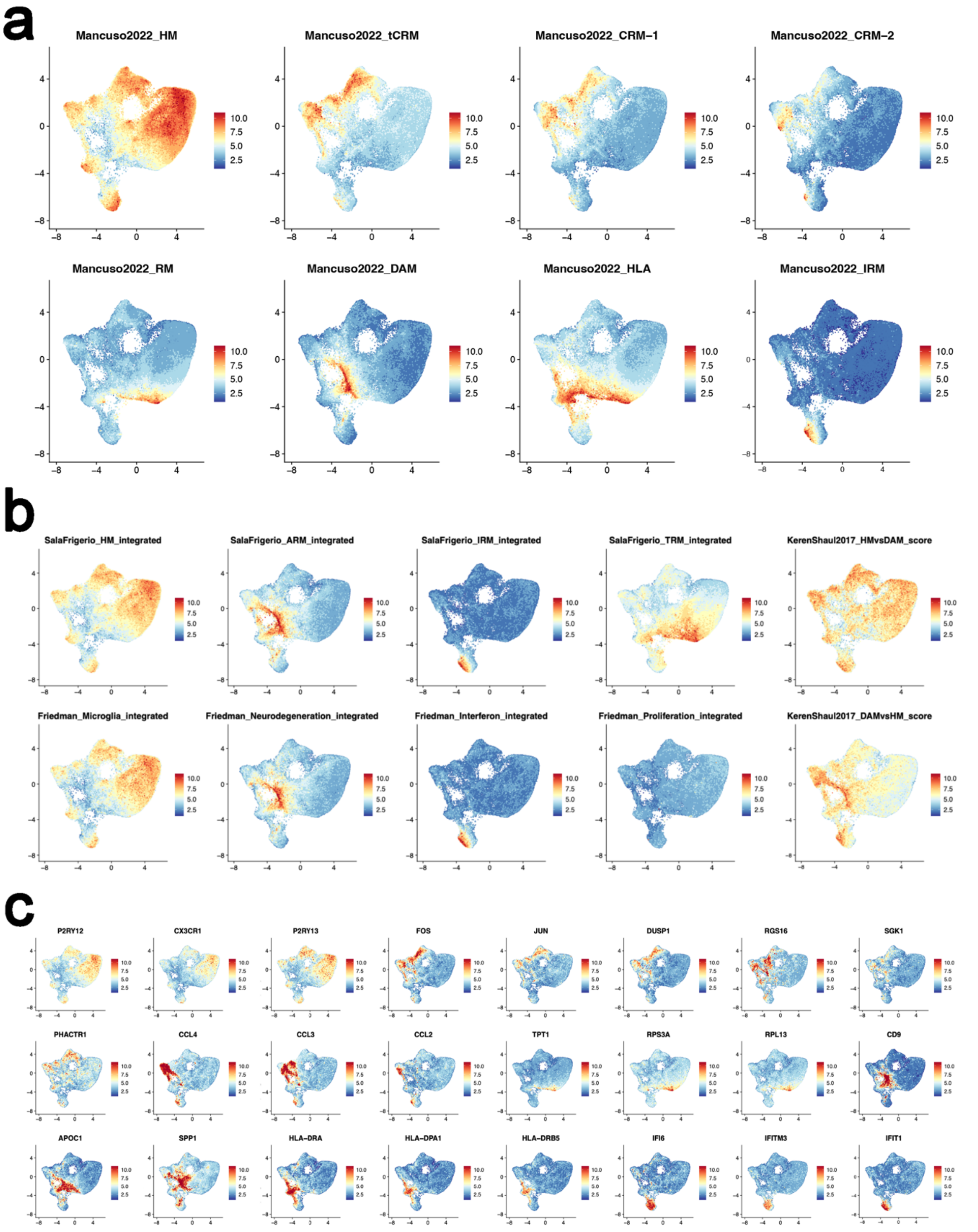

Supplementary Fig. 4. Extended cell state annotations. a, UMAP plots as in **Fig. 1a**, coloured by the combined level of expression of groups of genes that characterise distinct microglial states described in this study. **b,** UMAP plots as in Fig. 1a coloured by the combined level of expression of groups of genes that characterise distinct cell states described in mouse studies. **c,** Selected genes defining the different transcriptomic signatures shown in Fig. 1b (3 markers per cell state).

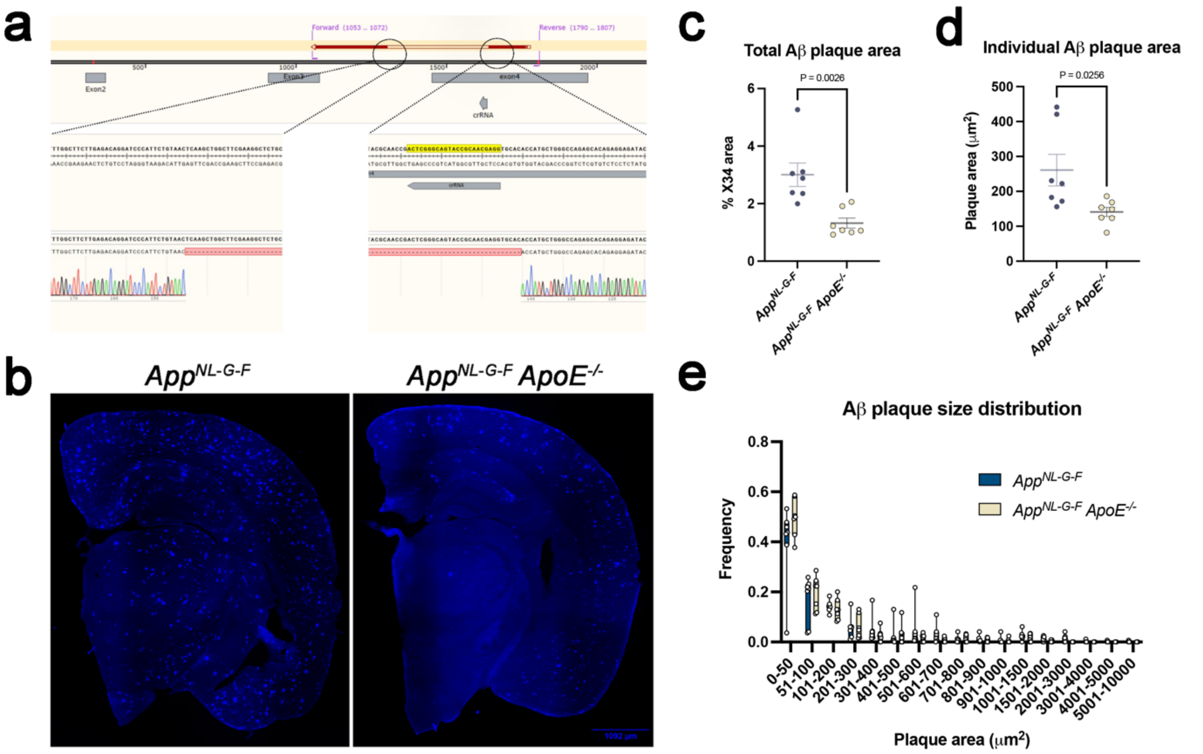

Supplementary Fig. 5 Reduction of amyloid-β plaque in ApoE$^{-/-}$ mice. a, Schematic representation of the generation of *App*$^{NL-G-F}$ *ApoE*$^{-/-}$ mice. The upper part represents the mouse *ApoE* allele on chromosome 7. Exons are indicated by grey boxes. The position of the start codon in exon 2 and the stop codon in exon 4 are indicated in red. The position of the primers used for genotyping are highlighted in pink. The sequence targeted by the Cas9 is labelled in yellow. The lower part shows the Sanger sequencing validation, with a confirmed deletion of 335bp spanning the last 148bp of intron 3 and first 187bp of exon 4. **b**, Representative coronal sections of *App*$^{NL-G-F}$ (n=7) and *App*$^{NL-G-F}$*xApoE*$^{-/-}$ (n=7) mice stained with X34. **c, d,** Quantification of (**c**) total and (**d**) individual Aβ plaque area (%X34 area) (unpaired t-test, ** $p<0.01$). (**e**) Size distribution of Aβ plaques expressed as frequency (y-axis) of plaques in different size intervals (x-axis). Bar plots in **c** and **d** represent mean +/- SEM, unpaired t-test, two-tailed, alpha = 0.05, significance set as P-value <0.05. Box plots in e are limited by lower and upper quartiles and midline indicates median; whiskers extend from the box to the smallest or largest value no further than 1.5*inter-quartile range.

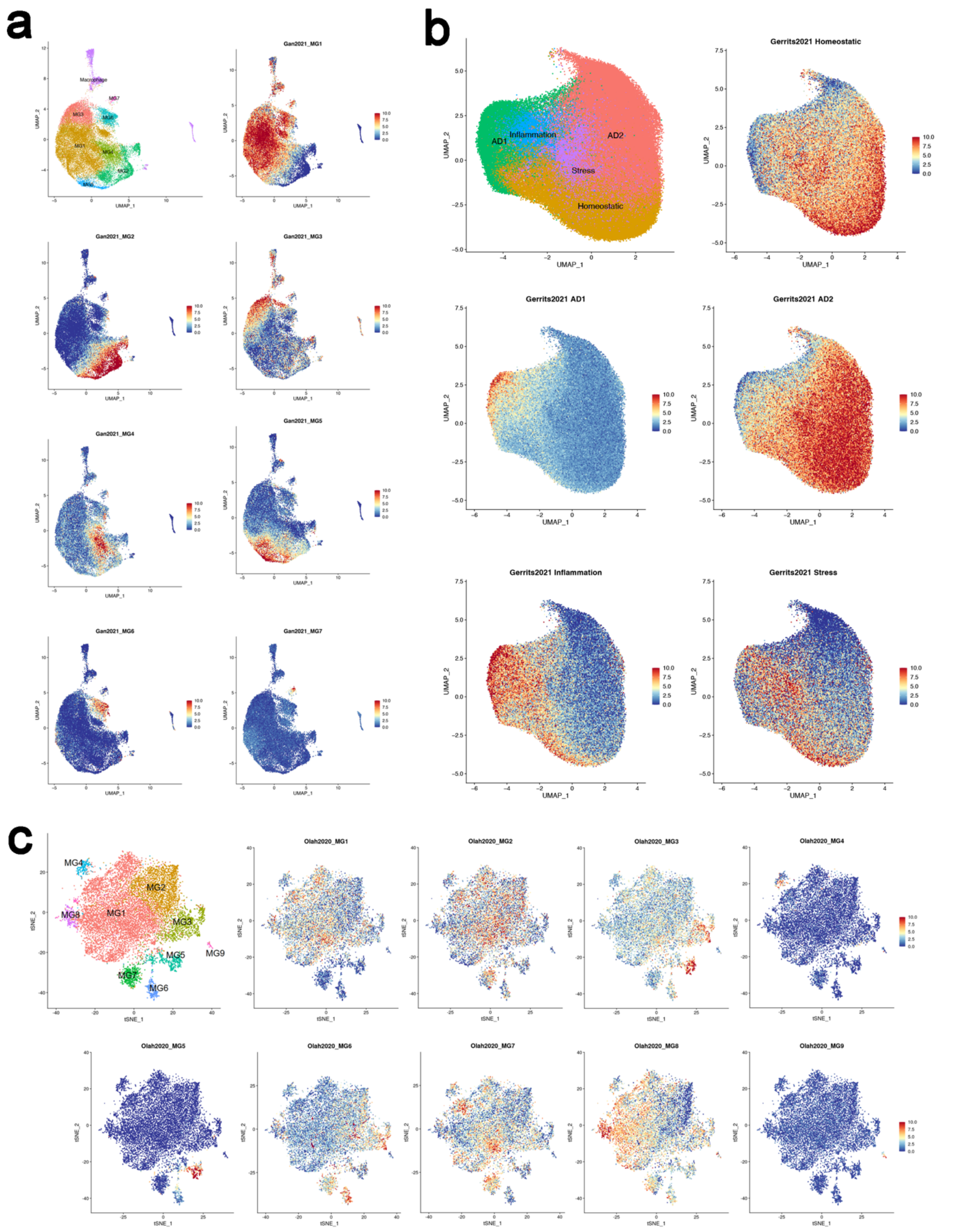

Supplementary Fig. 6. Extended analysis of the single microglia nuclei from human post-mortem brains. a, Seurat Module scores of sub-cluster genesets from supplementary data provided by Sayed et al.[35] ran against re-clustered Sayed et al.[35] single nuclei data following analysis workflow as described to reproduce annotations from the original study (see Methods, Sayed et al.[35]). **b,** Seurat Module scores of sub-cluster genesets from data provided by Gerrits et al.[20] ran against re-clustered Gerrits et al.[20] single nuclei data following analysis workflow as described to reproduce annotations from the original study (see Methods, Gerrits et al.[20]). **c,** Seurat Module scores of sub-cluster genesets from data provided by Olah et al.[36], ran against re-clustered Olah et al.[36] single cell data following analysis workflow as described to reproduce annotations from the original study (see Methods, Olah et al. 2020).

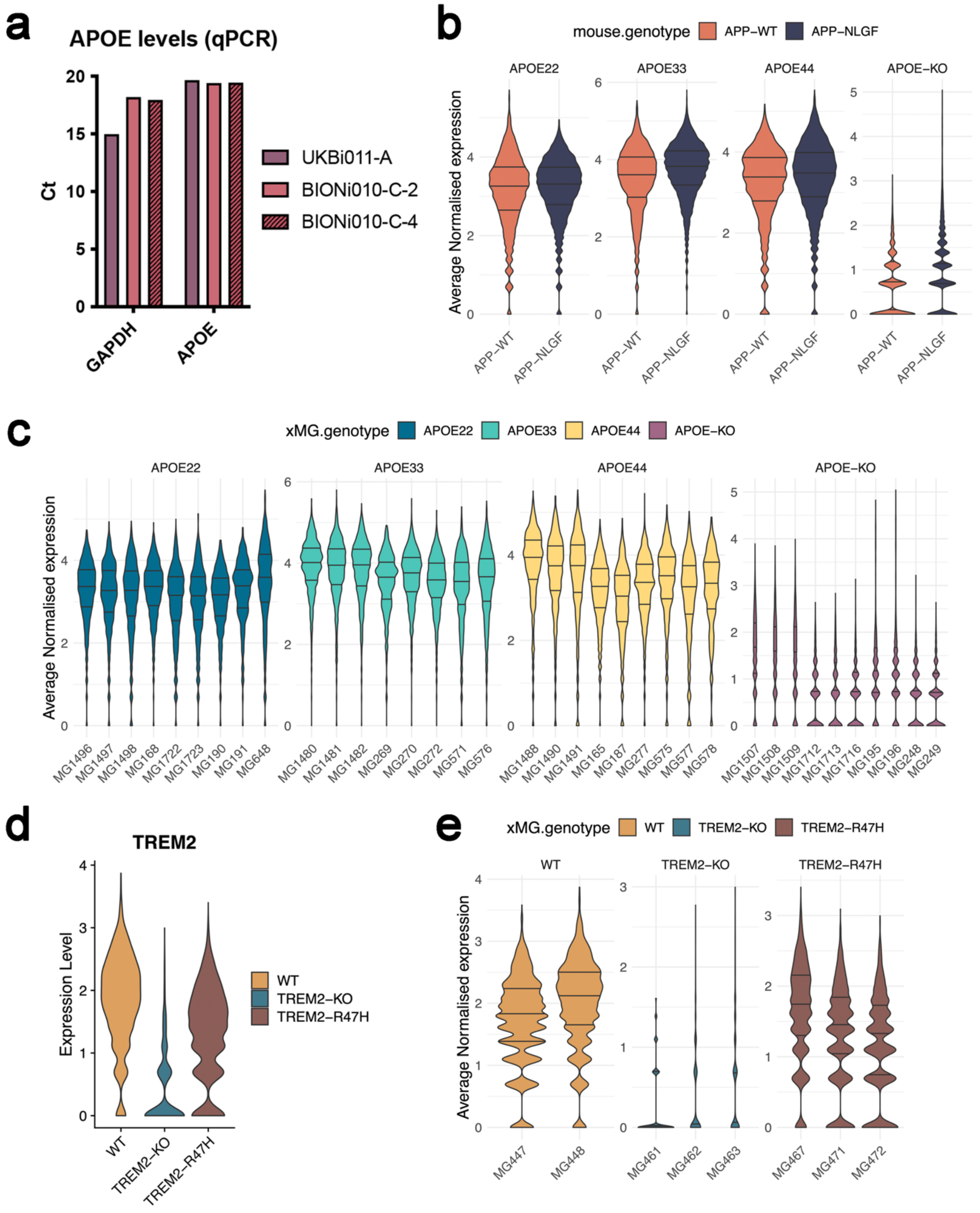

Supplementary Fig. 7. Levels of APOE expression across the different lines used in this study. a, Levels of expression of *APOE* and GAPDH in stem cells from UKBi011-A (iPSC-A, e4/e4), BIONi010-C-2 (iPSC-C, e3/KO) and BIONi010-C-4 (iPSC-3, e4/KO). **b,** Levels of APOE in transplanted microglia depicted per genotype, in both App^{WT} and $App^{NL\text{-}G\text{-}F}$. **c,** Levels of APOE in the transplanted cells for all the independent replicates used in this study. **d,** Levels of TREM2 in transplanted microglia depicted per genotype. **e,** Levels of APOE in the transplanted cells for all the independent replicates used in this study. Violin plots in **b, c** and **e** show the 25th, 50th and 75th percentiles with horizontal lines.
