# Supplementary material for: Xenografted human microglia display diverse transcriptomic states in response to Alzheimer’s disease-related amyloid-β pathology
Source: Nat Neurosci. 2024 Mar 27;27(5):886–900. doi: 10.1038/s41593-024-01600-y (PMC11089003; doi:10.1038/s41593-024-01600-y)
Supplement: Supplementary file 2 — Reporting Summary [file 41593_2024_1600_MOESM2_ESM.pdf]

nature portfolio

Corresponding author(s): Bart De Strooper and Renzo mancuso

Last updated by author(s): Jan 30, 2024

Reporting Summary

Nature Portfolio wishes to improve the reproducibility of the work that we publish. This form provides structure for consistency and transparency in reporting. For further information on Nature Portfolio policies, see our Editorial Policies and the Editorial Policy Checklist.

Statistics

For all statistical analyses, confirm that the following items are present in the figure legend, table legend, main text, or Methods section.

n/a	Confirmed	
☐	☒	The exact sample size (*n*) for each experimental group/condition, given as a discrete number and unit of measurement
☐	☒	A statement on whether measurements were taken from distinct samples or whether the same sample was measured repeatedly
☐	☒	The statistical test(s) used AND whether they are one- or two-sided<br>*Only common tests should be described solely by name; describe more complex techniques in the Methods section.*
☐	☒	A description of all covariates tested
☐	☒	A description of any assumptions or corrections, such as tests of normality and adjustment for multiple comparisons
☐	☒	A full description of the statistical parameters including central tendency (e.g. means) or other basic estimates (e.g. regression coefficient) AND variation (e.g. standard deviation) or associated estimates of uncertainty (e.g. confidence intervals)
☐	☒	For null hypothesis testing, the test statistic (e.g. *F*, *t*, *r*) with confidence intervals, effect sizes, degrees of freedom and *P* value noted<br>*Give P values as exact values whenever suitable.*
☒	☐	For Bayesian analysis, information on the choice of priors and Markov chain Monte Carlo settings
☒	☐	For hierarchical and complex designs, identification of the appropriate level for tests and full reporting of outcomes
☐	☒	Estimates of effect sizes (e.g. Cohen's *d*, Pearson's *r*), indicating how they were calculated

Our web collection on statistics for biologists contains articles on many of the points above.

Software and code

Policy information about availability of computer code

Data collection	The raw BCL files were demultiplexed and aligned by Cellranger (v.3.1.0) against the human genome database (hg19, Ensembl 87). Microscopy data was collected using Carl Zeis Microscopy V3.797. Flow cytometry data was collected using MACSQuantify™ Tyto® Software 1.0.
Data analysis	Raw count matrices were imported in R (v4.1.3) for data analysis. Single cell datasets were analyzed using the Seurat R package pipeline (v.4.0.1) The other packages and their versions used for the analyses of this study are reported as the result of SessionInfo() below. For specific statistical tests and visualizations, we also used GraphPad Prism v9.0, Python, R and Bioconductor. Flow cytometry data was analyzed using FCS Express 7. Microscopy data was analyzed using ImageJ.

For manuscripts utilizing custom algorithms or software that are central to the research but not yet described in published literature, software must be made available to editors and reviewers. We strongly encourage code deposition in a community repository (e.g. GitHub). See the Nature Portfolio guidelines for submitting code & software for further information.

Data

Policy information about availability of data

All manuscripts must include a data availability statement. This statement should provide the following information, where applicable:

- Accession codes, unique identifiers, or web links for publicly available datasets
- A description of any restrictions on data availability
- For clinical datasets or third party data, please ensure that the statement adheres to our policy

Data generated in this study are available at Gene Expression Omnibus (GEO) database with accession number GSE216999. Other datasets included in the manuscript can be found at GEO (Mancuso et al., GSE137444; Sala Frigerio et al., GSE127893; Hasselman et al., GSE133433; Gerrits et al., GSE148822; Sayed et al., GSE183068; Zhou et al., GSE140511; Keren-Shaul et al., GSE98969; Friedman et al., GSE89482).

Human research participants

Policy information about studies involving human research participants and Sex and Gender in Research.

Reporting on sex and gender	No human participants were used in this study
Population characteristics	Not applicable
Recruitment	Not applicable
Ethics oversight	Not applicable

Note that full information on the approval of the study protocol must also be provided in the manuscript.

Field-specific reporting

Please select the one below that is the best fit for your research. If you are not sure, read the appropriate sections before making your selection.

☒ Life sciences ☐ Behavioural & social sciences ☐ Ecological, evolutionary & environmental sciences

For a reference copy of the document with all sections, see nature.com/documents/nr-reporting-summary-flat.pdf

Life sciences study design

All studies must disclose on these points even when the disclosure is negative.

Sample size	Sample size was estimated based on previous experiments performed in the lab (Mancuso et al., 2019; Sala-Frigerio et al. 2019)
Data exclusions	We excluded from this dataset 6 mice that showed signs of infection, extremely low cell numbers and/or mice with the vast majority of cells mapping to one unique cell state.
Replication	The final high-quality microglia dataset consisted of 138,577 cells from 101 independent mice and 20 sequencing libraries.<br>For the analysis of human microglial phenotypes in response to amyloid beta, we used three independent cell lines across 39 mice.<br>For the analysis of the effect of plaques vs. soluble aggregates, we used two independent cell lines across 37 mice.<br>For the analysis of the effect of TREM2, we used one series of isogenic lines across 8 mice<br>For the assessment of the effect of APOE, we used two independent series of isogenic cell lines across 24 mice<br>For all statistical test and figures, each data point represents the single cell data set of one mouse.
Randomization	Experimental groups were ramdomised to avoid gender, litter and cage effects
Blinding	Investigators were blinded when performing all experiments

Reporting for specific materials, systems and methods

We require information from authors about some types of materials, experimental systems and methods used in many studies. Here, indicate whether each material, system or method listed is relevant to your study. If you are not sure if a list item applies to your research, read the appropriate section before selecting a response.

Materials & experimental systems

n/a	Involved in the study
	☒ Antibodies
	☒ Eukaryotic cell lines
☒	☐ Palaeontology and archaeology
	☒ Animals and other organisms
☒	☐ Clinical data
☒	☐ Dual use research of concern

Methods

n/a	Involved in the study
☒	☐ ChIP-seq
	☒ Flow cytometry
☒	☐ MRI-based neuroimaging

Antibodies

Antibodies used

PE-Pan-CD11b (1:50, Miltenyi, Cat#130-113-806)
Viability dye (1:2000, eFluor 780, Thermo Fisher Scientific, Cat#65-0865-14)
APC-hCD45 (1:50, BD Biosciences, Cat#555485)
BV421-mCD45 (1:500, BD Biosciences, Cat#563890)
Anti-human CD9 (1:500, Biolegend, Cat#312102)
Anti-human HLA DR+DP+DQ [CR2/43] (1:200, Abcam, Cat#ab7856)
Anti-human P2RY12 (1:1000, Sigma Aldrich, Cat#HPA014518)
Anti-human FHT1 (1:500, Invitrogen, Cat#PA5-19058)
Donkey anti-rabbit (H+L) Alexa Fluor 647 (1:1000, Thermo Fisher, Cat#A31573)
Donkey anti-mouse (H+L) Alexa Fluor 594 (1:1000, Thermo Fisher, Cat#A21203)
Donkey anti-goat (H+L) Alexa Fluor 594 (1:1000, Thermo Fisher, Cat#A32758)
Alexa Fluor 594 Tyramide SuperBoost, goat anti-mouse IgG (1:1000, Thermo Fisher, Cat#B40915)
TotalSeqTM-A cell hashing antibodies (1:500, Biolegend)
Mouse FcR blocker (1:10, Miltenyi, Cat#130-092-575)
X34 staining solution (10µM Sigma-Aldrich)

Validation

PE-Pan-CD11b: validated in splenocytes from BALB/c mice were stained with CD11b antibodies or with the corresponding REA Control antibodies.
APC-hCD45: validated on human peripheral blood lymphocytes, stained with either APC Mouse IgG1, or κ isotype control.
BV421-mCD45: validated on mouse splenic leucocytes preincubated with Purified Rat Anti-Mouse CD16/CD32 antibody and then stained with either BD Horizon™ BV421 Rat IgG2b or κ Isotype Control.
Anti-human CD9: validated on the BT474 breast cancer cell line.
Anti-human HLA DR+DP+DQ [CR2/43]: validated on sections of formalin-fixed paraffin-embedded normal human tonsil or colorectal tissue.
Anti-human P2RY12: validated on sections of human cerebral cortex and liver tissue.
Anti-human FHT1: validated in HeLa cells and human kidney tissue.

Eukaryotic cell lines

Policy information about cell lines and Sex and Gender in Research

Cell line source(s)

Name of line / Genotype / Source / Citation
UKBi011-A / APOE e4/e4 / Bioneer,EBiSC / RRID:CVCL_LE34
UKBi011-A-1 / APOE KO/KO / Bioneer, EBiSC / RRID:CVCL_RM82
UKBi011-A-2 / APOE e2/e2 / Bioneer, EBiSC / RRID:CVCL_VN45
UKBi011-A-3 / APOE e3/e3 / Bioneer, EBiSC / RRID:CVCL_RX83
BIONi010-C-2 / APOE e3/KO / Bioneer, EBiSC / RRID:CVCL_II81
BIONi010-C-3 / APOE KO/KO / Bioneer, EBiSC / RRID:CVCL_II82
BIONi010-C-4 / APOE e4/KO / Bioneer, EBiSC / RRID:CVCL_II83
BIONi010-C-6 / APOE e2/KO / Bioneer, EBiSC / RRID:CVCL_II85
H9 (WA09) / APOE e3/e4 / WiCell Research Institute / RRID:CVCL_9773
H9-iCas9 / APOE e3/e4 / VIB-Center for Brain and Disease Research / Not cited
H9-TREM2-/- / TREM2-/- / KUL Stem cell Institute / Claes et al., 2019
H9-TREM2R47H / TREM2R47H / KUL Stem Cell Institute / Claes et al., 2019

Authentication

The following cell lines were authenticated by the providers by Karyotyping and whole genome sequencing, and have been tested for pluripotency
- H9 (WAe009-A) - https://hpscreg.eu/cell-line/WAe009-A
- BIONi010-C and subsequent genetic modifications - https://hpscreg.eu/cell-line/BIONi010-C
- UKBi011-A and subsequent genetic modifications - https://hpscreg.eu/cell-line/UKBi011-A
The H9-iCas9, H9-TREM2-/- and H9-TREM2R47H lines were tested for chromosomal aberrations and confirmed for pluripotency by qPCR and immunocytochemistry.

Mycoplasma contamination

All the lines used were regularly tested negative for mycoplams

Commonly misidentified lines
(See ICLAC register)

None of the cell lines used in this study is known to be cross-contaminated or otherwise misidentified, and is not listed in the Register of Misidentified Cell Lines from ICLAC

Animals and other research organisms

Policy information about studies involving animals; ARRIVE guidelines recommended for reporting animal research, and Sex and Gender in Research

Laboratory animals	We used three mouse strains in this study:<br>- AppNL-G-F Rag2-/- IL2rg-/- hCSF1-KI<br>- AppHu/Hu Rag2-/- IL2rg-/- hCSF1-KI<br>- AppNL-G-F Rag2-/- IL2rg-/- hCSF1-KI / ApoE-/-<br>All strains were kept in a C57Bl/6xBalbC background. Mice had access to food and water ad libitum and were housed with a 14/10 h light-dark cycle at 21°C and 32% humidity, in groups of two to five animals.
Wild animals	No wild animals were used
Reporting on sex	Experimental groups were balanced in terms of the sex of the mice
Field-collected samples	No field-collected samples were used in this study
Ethics oversight	Animal experiments were approved by the local Ethical Committee of Laboratory Animals of the KU Leuven (government licence LA1210579 ECD project number P177/2017) following local and EU guidelines.

Note that full information on the approval of the study protocol must also be provided in the manuscript.

Flow Cytometry

Plots

Confirm that:

☒ The axis labels state the marker and fluorochrome used (e.g. CD4-FITC).

☒ The axis scales are clearly visible. Include numbers along axes only for bottom left plot of group (a 'group' is an analysis of identical markers).

☒ All plots are contour plots with outliers or pseudocolor plots.

☒ A numerical value for number of cells or percentage (with statistics) is provided.

Methodology

Sample preparation	Mic were sacrificed with an overdose of sodium pentobarbital and immediately perfused with ice-cold 1x DPBS (Gibco, Cat#14190-144) supplemented with 5U of heparin (LEO). After perfusion, 1 hemisphere of each mouse brain without cerebellum and olfactory bulbs was placed in FACS buffer (1x DPBS, 2% FCS and 2 mM EDTA) + 5 µM Actinomycin D (ActD, Sigma, Cat#A1410-5MG) for transcriptomics. Brains were mechanically and enzymatically dissociated using Miltenyi neural tissue dissociation kit P (Miltenyi, Cat#130-092-628) supplemented with 5 µM ActD. Next, samples were passed through a 70 µm strainer (BD2 Falcon), washed in 10 ml of ice-cold FACS buffer + 5 µM ActD and spun at 300g for 15 minutes at 4°C. Note that 5 µM ActD was kept during collection and enzymatic dissociation of the tissue to prevent artificial activation of human microglia during the procedure as previously reported12. ActD was removed from the myelin removal step to prevent toxicity derived from long-term exposure. Following dissociation, myelin was removed by resuspending pelleted cells into 30% isotonic Percoll (GE Healthcare, Cat#17-5445-02) and centrifuging at 300g for 15 minutes at 4°C. Accumulating layers of myelin and cellular debris were discarded and Fc receptors were blocked in FcR blocking solution (1:10, Miltenyi, Cat#130-092-575) in cold FACS buffer for 10 minutes at 4°C. Next, cells were washed in 5 ml of FACS buffer and pelleted cells were incubated with the following antibodies: PE-Pan-CD11b (1:50, Miltenyi, Cat#130-113-806), BV421-mCD45 (1:500, BD Biosciences, Cat#563890), APC-hCD45 (1:50, BD Biosciences, Cat#555485), TotalSeqTM-A cell hashing antibodies (1:500, Biolegend) and viability dye (1:2000, eFluor 780, Thermo Fisher Scientific, Cat#65-0865-14) in cold FACS buffer during 30 minutes at 4°C. After incubation, cells were washed, and the pellet was resuspended in 500 µl of FACS buffer and passed through 35 µm strainer prior sorting.
Instrument	MACSQuant Tyto
Software	MACSQuantify™ Tyto® Software 1.0 and FCS express 7
Cell population abundance	As shown in Figure S1, both mouse host (CD11b+ mCD45+) and human transplanted microglia (CD11b+ hCD45+) are two clear distinct populations. This study did not aim to quantify these populations by flow cytometry, but rather purify them for downstream single cell RNA sequencing analysis.
Gating strategy	BSC and SSC were used to filter debri and doublet discrimination. e780 (Thermo Fisher) was used as a viability marker. All analyses were performed on viable singlets. Full gating strategy is shown in Figure S1.

☒ Tick this box to confirm that a figure exemplifying the gating strategy is provided in the Supplementary Information.
